# Cell-Free Reaction System for ATP Regeneration from d-Fructose

**DOI:** 10.1021/acssynbio.4c00877

**Published:** 2025-03-27

**Authors:** Franziska Kraußer, Kenny Rabe, Christopher M. Topham, Julian Voland, Laura Lilienthal, Jan-Ole Kundoch, Daniel Ohde, Andreas Liese, Thomas Walther

**Affiliations:** † Chair of Bioprocess Engineering, Institute of Natural Materials Technology, TU Dresden, Bergstraße 120, 01062 Dresden, Germany; ‡ Molecular Forces Consulting, 24 Avenue Jacques Besse, 81500 Lavaur, France; § Institute of Technical Biocatalysis, Hamburg University of Technology, Denickestr. 15, 21073 Hamburg, Germany

**Keywords:** in vitro biocatalysis, ATP cofactor regeneration, phosphoketolase, acetyl phosphate synthesis, molecular modeling, semirational enzyme engineering

## Abstract

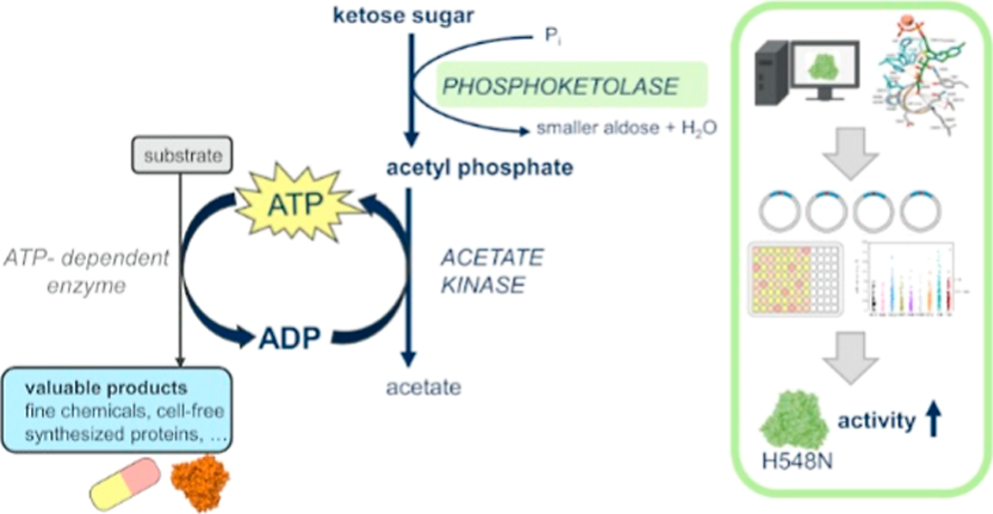

Adenosine triphosphate (ATP)-dependent *in vitro* bioprocesses, such as cell-free protein synthesis and the production of phosphorylated fine chemicals, are of considerable industrial significance. However, their implementation is mainly hindered by the high cost of ATP. We propose and demonstrate the feasibility of a cell-free ATP regeneration system based on the *in situ* generation of the high-energy compound acetyl phosphate from low-cost d-fructose and inorganic phosphate substrates. The enzyme cascade chains d-fructose phosphoketolase, d-erythrose isomerase, d-erythrulose phosphoketolase, and glycolaldehyde phosphoketolase activities theoretically enabling production of 3 mol ATP per mol of d-fructose. Through a semirational engineering approach and the screening of nine single-mutation libraries, we optimized the phosphoketolase (PKT) from *Bifidobacterium adolescentis*, identifying the improved variant Bad.F6Pkt H548N. This mutant exhibited a 5.6-fold increase in d-fructose activity, a 2.2-fold increase in d-erythrulose activity, and a 1.3-fold increase in glycolaldehyde activity compared to the wild-type enzyme. The Bad.F6Pkt H548N mutant was initially implemented in a cell-free reaction system together with an acetate kinase from *Geobacillus stearothermophilus* and a glycerol kinase from *Cellulomonas* sp. for the production of glycerol-3 phosphate from ADP and glycerol. We demonstrated the feasibility of ATP regeneration from 25 mM d-fructose with a stoichiometry of 1 mol of ATP per mol of C_6_ ketose. Subsequently, the reaction system was enhanced by incorporating d-erythrose isomerase activity provided by a l-rhamnose isomerase from *Pseudomonas stutzeri*. In the complete system, the ATP yield increased to 2.53 mol mol_fructose_^–1^ with a maximum productivity of 7.2 mM h^–1^.

## Introduction

A substantial number of enzymatic reactions rely on the cofactor adenosine triphosphate (ATP), which is used as a source of energy or phosphoryl donor. Consequently, the industrial significance of ATP-dependent *in vitro* bioprocesses is considerable, particularly with regard to the cell-free synthesis of proteins,^1−3^ phosphorylated fine chemicals, pharmaceuticals,^4−6^ and food additives.^7^ Given the high financial burden associated with the addition of ATP in stoichiometric quantities, the implementation of a cost-efficient cofactor regeneration system is required for the application of ATP-dependent cell-free biotransformations.

Several methods are in use for regenerating ATP. In small scale reaction systems (μL to mL scale^8−10^), the most common regeneration systems involve the transfer of a phosphoryl group from a high-energy compound such as phosphoenolpyruvate,^11^ acetyl-phosphate (AcP),^12^ or creatine phosphate^13^ to adenosine diphosphate (ADP) catalyzed by a kinase. However, high costs and low stability of the phosphate donors render these ATP regeneration methods unsuitable for biosyntheses on larger scales (mL to L).

In industrial-scale applications, inexpensive polyphosphate (PolyP) is frequently used as a high-energy phosphate donor which can be used to recycle ATP by employing a polyphosphate kinase.^14,15^ However, coupling of polyphosphate-based ATP regeneration to the synthesis of nonphosphorylated end products (e.g., cell-free synthesized proteins) results in the accumulation of inorganic phosphate. This may lead to significant inhibition of the product synthesis due to complexation and precipitation of essential ions which is, therefore, prohibitive for accumulating high product concentrations.^1^ To circumvent this problem, ATP-yielding enzyme cascades emanating from the nonphosphorylated substrates pyruvate and glutamate have been conceived. Pyruvate can be converted to AcP (and CO_2_) using phosphorylating pyruvate oxidase (POX).^1,6,7^ Glutamate was shown to fuel the respiratory chain present in cell membranes of the cell debris, thus enabling ATP recycling via plasma-membrane ATP synthase.^16^ While these methods prevent accumulation of inhibiting phosphate concentrations, they both rely on the presence of oxygen. Hence, these reaction systems need to be aerated, which causes severe problems due to excessive foaming of the protein solution. This complicates process design and ultimately results in higher costs.

Besides POXs, phosphoketolases (PKTs) are also capable of generating the high-energy phosphoryl donor AcP. Naturally, PKTs cleave the C2–C3 bond of the phosphorylated sugars xylulose 5-phosphate (EC 4.1.2.9) and/or fructose 6-phosphate (F6P) (EC 4.1.2.22) in a thiamine diphosphate (TPP) dependent reaction releasing aldose phosphate as product. Subsequent dehydration and phosphorolysis produces AcP.^17−19^ However, the phosphate group present in the substrate does not participate in the reaction mechanism.^20^ Thus, if PKT could be applied to produce AcP from nonphosphorylated ketose sugars, the cost of ATP regeneration would be significantly reduced without the need for aeration. Interestingly, synthesis of AcP from the nonphosphorylated sugars d-erythrulose and glycolaldehyde was recently demonstrated corroborating this idea.^21,22^

**Figure 1:**
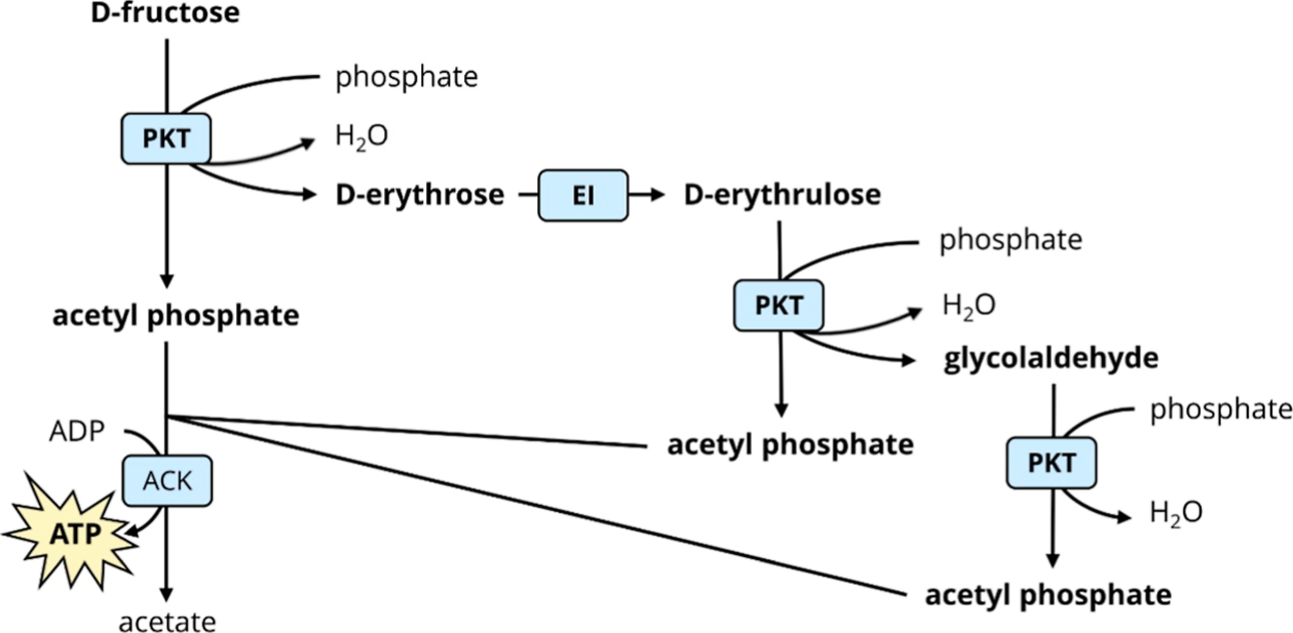
Enzymatic cascade for ATP regeneration from d-fructose. Abbreviations: PKT—phosphoketolase, EI—d-erythrose isomerase, ACK—acetate kinase, ADP—adenosine diphosphate.

Based on these considerations, we propose a reaction cascade that first converts d-fructose to d-erythrose and AcP in a PKT-catalyzed reaction. This cascade can be complemented by a d-erythrose isomerase, which converts d-erythrose to d-erythrulose, thus enabling the production of two additional AcP molecules from erythrulose and glycolaldehyde in PKT-dependent reactions (see above). AcP is then used to regenerate ATP from ADP using acetate kinase (Figure 1). The complete cascade has a theoretical stoichiometry of three mol of ATP per mole of fructose. Its thermodynamic feasibility is witnessed by the highly negative standard Gibbs energy of the PKT reactions (Δ_r_*G*′° = −56.3 kJ mol^–1^, calculated using online eQuilibrator^23^).

In this study, we set out to implement this enzyme cascade. We first carried out semirational engineering of the PKT from *Bifidobacterium adolescentis* (Bad.F6Pkt), which yielded the Bad.F6Pkt H548N variant that had a 5.6, 2.2, and 1.3-fold increased activity on the nonphosphorylated substrates d-fructose, d-erythrulose, and glycolaldehyde, respectively, compared to the wild-type (WT) enzyme. This variant was applied together with an acetate kinase and a glycerol kinase for producing glycerol-3 phosphate from ADP and glycerol in a cell-free reaction system. We demonstrated the feasibility of ATP regeneration from d-fructose with a stoichiometry of 1 mol of ATP per mol of fructose. Furthermore, we showed that addition of an erythrose isomerase to the reaction system increased the ATP yield to 2.53 mol mol_fructose_^–1^.

## Results

### Wild-Type PKT from *Bifidobacterium adolescentis* Exhibits Significant Activity on d-Fructose

Due to their high natural activity on F6P, four PKT enzymes from different organisms, namely *B. adolescentis*, *Bifidobacterium breve* and *Clostridium acetobutylicum*,^20,24−27^ were assessed for their ability to convert the nonphosphorylated substrate d-fructose to AcP. To this end, the corresponding genes were cloned into the pET-28(a)+ expression vector to add a N-terminal His-tag. The proteins were expressed using the *Escherichia coli* BL21 (DE3) or Rosetta (DE3) plysS strain (Table S7), purified, and characterized for their activity on d-fructose using the colorimetric hydroxamate assay, which quantifies the AcP that is released in the PKT reaction.^28,29^ To quantify the substrate affinity of the enzymes, fructose concentration was varied between zero and 300 mM. The PKT from *B. adolescentis* (Bad.F6Pkt) exhibited the highest specific activity with 0.11 U mg^–1^ (observed at 300 mM fructose), followed by the enzyme from *B. breve* strain 203 (Bb.Xfp) with 0.03 U mg^–1^ (Table S3). Within the tested fructose concentration range, no substrate saturation was observed for any of the four candidate enzymes. Given that Bad.F6Pkt demonstrated the highest specific activity on d-fructose among the tested enzymes, it was selected as the template for engineering a PKT with enhanced activity on this nonphosphorylated substrate.

### Identification of Target Residue Positions in Bad.F6Pkt for Mutagenesis

In order to increase activity and affinity of PKT on the non-natural substrate d-fructose by enzyme engineering, we sought to identify target positions for mutation from inspection of a modeled complex of the wild type Bad.F6Pkt enzyme with the TPP-F6P linear covalent adduct, aided by inferred tolerated position-dependent residue variation from analysis of a PKT protein family multiple sequence alignment (MSA) (see Methods).

**Figure 2:**
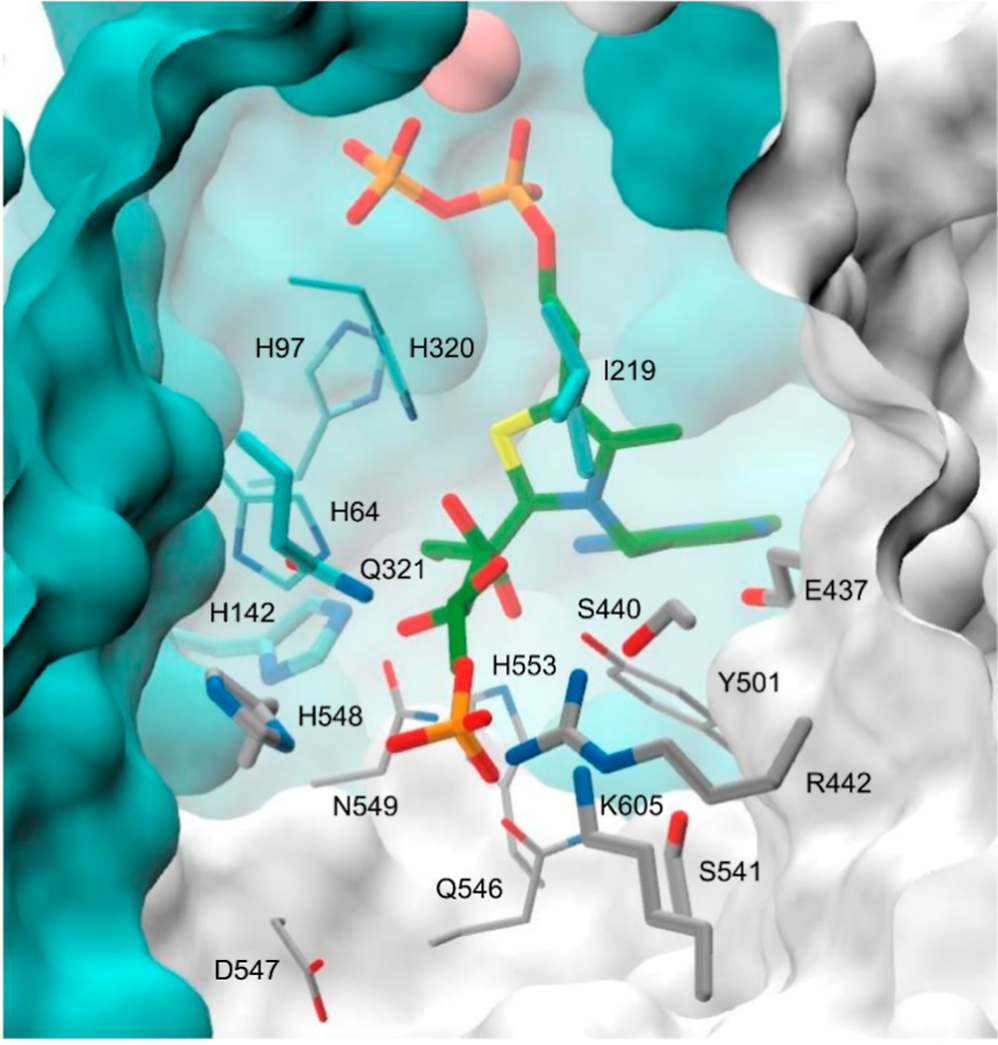
TPP fructose-6-P adduct in the *B. adolescentis* PKT active-site with target positions for site-directed mutagenesis. The TPP-fructose-6-P (T6F) covalent adduct is shown in stick representation with green colored carbon atoms. Side-chain carbon atoms at active site residue positions catalogued in Table S1 in the PP- and PYR-domains at the PKT homodimer interface are, respectively, colored cyan and gray. Side-chains at a subset of target positions selected for saturated single-site mutagenesis are shown as thicker sticks. Other atom types are colored according to the chemical elements: oxygen—red, nitrogen—blue, sulfur—yellow, phosphorus—orange. The Mg^2+^ ion bound to the pyrophosphate moiety of TPP is depicted as a solid van der Waals sphere (pink). The background apoenzyme solvent excluded surface, calculated following the removal the all PKT residues in the first contact shell with TPP-T6F, is colored according to protein domain: PP, cyan; PYR, gray.

The Bad.F6Pkt enzyme shares ≥85% sequence identity with the PKT enzymes from *B. breve* and *Bifidobacterium longum* (Figure S1), for which crystal structures have previously been reported.^20,30,31^ The X-ray structure of *B. breve* PKT H320A mutant (PDB entry 3AHI,^20^) was used as a structural template to build a model complex of *B. adolescentis* PKT (UniProtKB entry A1A185) with Mg-TPP and the open-chain form of d-fructose-6-phophate (F6P). A total of 17 putative residue positions for study by saturation mutagenesis were identified in the first contact shell with the TPP-F6P (T6F) covalent adduct in the catalytic center and extended sugar substrate binding channel at the PKT homodimer interface (Figure 2 and Table S3). Crystallographic^20,30^ and theoretical computational^32^ studies have identified five histidine residue positions (64, 97, 142, 320, and 553) as potentially playing key mechanistic roles in the formation of the α,β-dihydroxyethyl TPP (DHETPP) intermediate and its subsequent dehydration. These residue positions are accordingly all found to be heavily conserved among PKT enzymes with low relative Shannon sequence entropy (H_*x*_) values in the range of 0.02 to 0.05 (Table S3). However, although H142 has been implicated as a putative proton donor in the dehydration of DHETPP, Yang et al.^22^ have recently demonstrated that affinity for short ketoses can be enhanced by mutation. The inorganic phosphate (Pi) substrate binding subsite in Bad.F6Pkt comprises H64, H320, Q321, Y501, and N549 polar residues. Q321 is the least conserved residue position in the Pi binding site (H_*x*_ = 0.28), and mutation to alanine resulted only in a minor effect on the affinity of PKT from *B. breve* for inorganic phosphate.^20^

Guided by available experimental evidence and theoretical H_*x*_ measures of inferred mechanistic importance, summarized in Table S3, 9 of the 17 identified active site residues were chosen as target positions for altering substrate specificity without undue considered incurred risk of activity loss: H142, I219, Q321, E437, S440, R442, S541, H548, and K605. The mutated residue positions, highlighted in Figure 2, are for the most part orientated toward the C6 terminal hydroxyl group of the nonphosphorylated fructose target substrate and the vacant space occupied by the cognate F6P phosphate group.

### Single Mutations in Positions Q321, S541, H548, and K605 Increase Bad.F6Pkt Activity on d-Fructose

**Figure 3:**
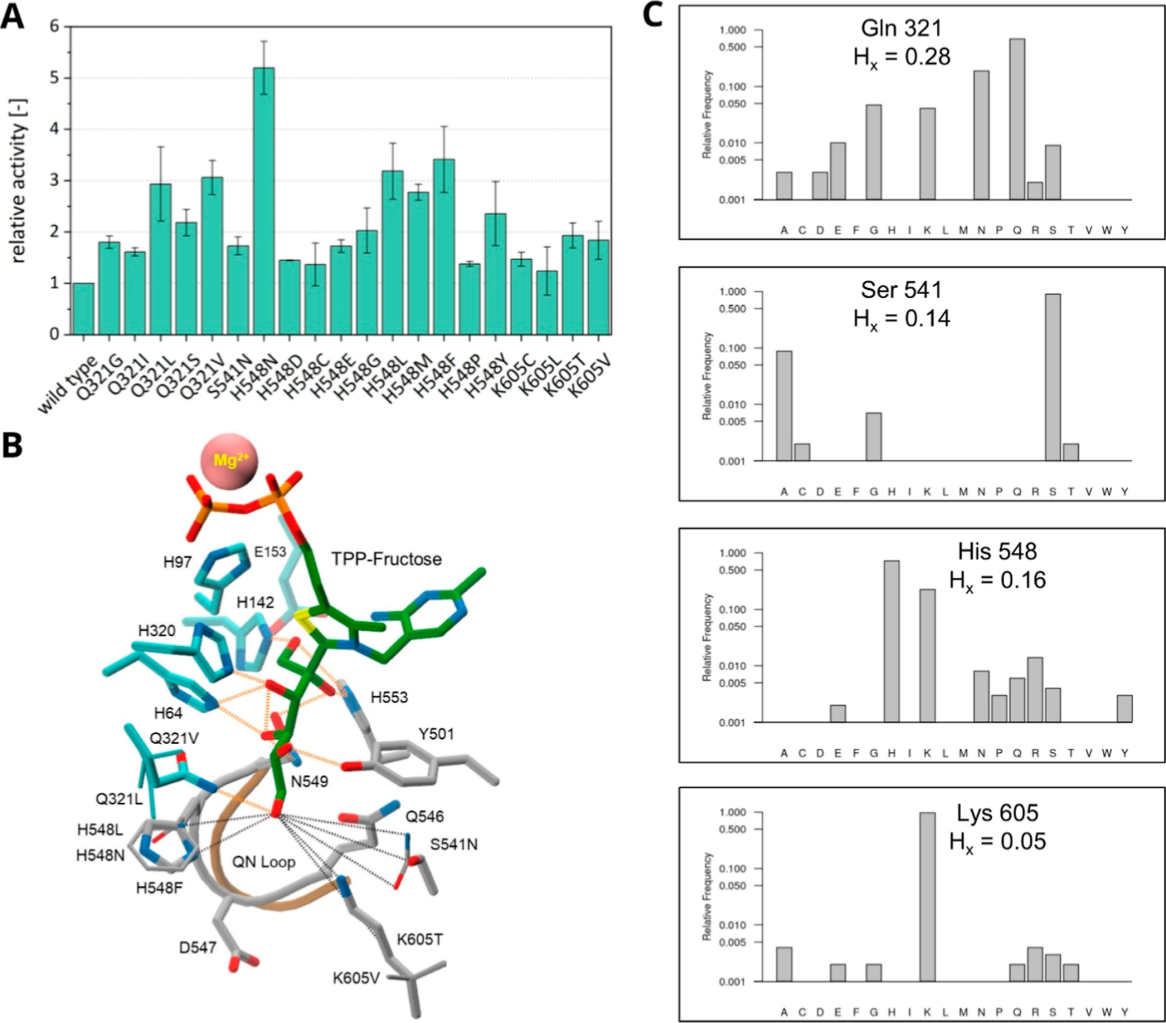
Single mutants of Bad.F6Pkt with increased activity on fructose. (A) Relative d-fructose activity of beneficial Bad.F6Pkt single mutants. After identification from the screening of single mutation libraries, beneficial variants were produced in shake flasks, purified and their specific activity determined at pH 6.5, 37 °C in the presence of 200 mM d-fructose. Relative activity was determined as the ratio of the specific activities of the mutant and WT enzymes. Error bars indicate deviation of the mean (*n* ≥ 2). (B) WT and mutant Bad.F6Pkt residue side-chain interactions with TPP-fructose. The TPP-fructose covalent adduct is shown in stick representation with green colored carbon atoms and the bound Mg^2+^ ion as a solid pink colored van der Waals sphere. Backbone ribbon overlays of the PYR domain Q546-N549 (QN) loop in the “closed” (active) and “open” states, abstracted from the Cryo-EM structure of *B. longum* Pkt complex with TPP (PDB entry 6LXV), are, respectively, depicted in gray and brown. Residue side-chains are shown in stick form, colored as described in the Figure 2 legend. Model built side-chains of selected single mutants, for which kinetic parameters are provided in Table 1, are represented as narrow sticks. Hydrogen bonds, defined by the PANORAMA structural analysis program^33^ according to standard geometric criteria, are shown in orange. Longer (>3.5 Å) range interactions between side-chain hydrogen bonding functional group atoms and the open-chain d-fructose C6 hydroxyl oxygen atom are depicted as dashed black line vectors. (C) MSA residue frequency profiles at selected mutated residue positions.

We next set out to characterize the impact of amino acid replacements in each of the identified target positions. To this end, the individual positions were mutated by saturating PCR, thus obtaining nine single-site saturated libraries. The resulting plasmids were transformed into *E. coli* Rosetta (DE3) plysS and plated on LB agar plates. Random distribution of mutations was verified by sequencing at least 5 individual clones from each target position library. Subsequently, 110 clones of each mutant library were picked and expressed in deep well plates. Fructose-specific PKT activity of the clones was assayed in microtiter plates directly in crude protein extracts obtained by lysing the cells. A total of 39 positive hits (defined as an activity of three standard deviations higher than the mean of the wild type activity) were identified in seven of the nine tested positions (Figure S4). These clones were sequenced, individually expressed in shake flasks, and purified to verify their fructose-specific PKT activity using the isolated proteins. This analysis yielded 20 variants in the four positions Q321, S541, H548, and K605, which exhibited at least 24% higher activity on the C_6_ ketose compared to the wild type Bad.F6Pkt (Figure 3A). The replacement of the WT residue in position Q321 by hydrophobic amino acids (V, L, and I) resulted in an increase in d-fructose activity, while in position S541, only one amino acid substitution (S541N) demonstrated a beneficial effect. In position K605, either small nonpolar (V, L) or polar amino acids (C, T) improved activity on the nonphosphorylated target substrate. Nine Bad.F6Pkt variants with residues of various physical properties in position H548 exhibited at least 37% higher fructose activity than the WT enzyme. The highest activity on d-fructose was achieved by replacing the histidine in position 548 with asparagine, resulting in a 5.6-fold increase in *v*_max_ compared to the WT enzyme (Table 1).

**Table 1:** Kinetic Parameters of Bad.F6Pkt Wild Type and Selected Variants^b^

	fructose 6-phosphate				d-fructose		
Bad.F6Pkt	*v*_max_ [U mg^–1^]	*K*_M_ [mM]	*K*_i_ [mM]	*k*_cat_/*K*_M_ [M^–1^ s^–1^]	*v*_max_^a^ [U mg^–1^]	*K*_M_ [mM]	*k*_cat_/*K*_M_ [M^–1^ s^–1^]
wild type	7.37 (±0.29)	10.52 (±0.26)	-	1082 (±66)	0.11 (±0.03)	n.sat.	n.d.
Q321L	0.61 (±0.02)	7.87 (±0.47)	202.40 (±16.90)	119 (±10)	0.27 (±0.07)	n.sat.	n.d.
Q321V	0.35 (±0.08)	0.54 (±0.07)	-	1006 (±90)	0.26 (±0.04)	n.sat.	n.d.
S541N	5.64 (±0.22)	118.92 (±22.92)	28.16 (±0.27)	77 (±18)	0.19 (±0.06)	n.sat.	n.d.
H548N	6.32 (±0.38)	10.31 (±0.31)	68.38 (±0.24)	945 (±29)	0.62 (±0.08)	n.sat.	n.d.
H548L	0.49 (±0.07)	9.61 (±0.69)	79.07 (±9.13)	78 (±5)	0.26 (±0.02)	n.sat.	n.d.
H548F	3.43 (±0.11)	8.64 (±1.93)	69.31 (±5.79)	650 (±165)	0.34 (±0.02)	n.sat.	n.d.
K605T	0.16 (±0.01)	28.50 (±5.29)	-	9 (±1)	0.20 (±0.04)	n.sat.	n.d.

a If substrate saturation was not observed within the concentration range tested, specific activity in the presence of the highest tested substrate concentration of 300 mM was defined as *v*_max_.

b Experiments were carried out at pH 6.5, 37 °C in the presence of 50 mM inorganic phosphate. Experimental data were fitted to the Michaelis–Menten or substrate inhibition model to determine kinetic parameters (curve fitting tool, MATLAB R2023b). n.sat.—no substrate saturation observed, n.d.—not determined due to unknown *K*_M_. Deviation of the mean is given in brackets (*n* ≥ 2).

The best Bad.F6Pkt variants in each of the four positions were analyzed regarding their kinetic parameters for both the natural and target substrates (Table 1). None of the tested variants exhibited saturating activity at fructose concentrations of up to 300 mM, thus, showing that the Bad.F6Pkt H548N enzyme was the most efficient enzyme. Mutations illustrated in Figure 3B that enhanced fructose activity inversely displayed significantly impaired catalytic efficiency for the natural substrate F6P. This result was not surprising since K605, H548, S541, and Q321 are involved in recognition of the phosphate moiety of the F6P substrate.^20,30^ PKT family MSA residue frequency profiles reveal particularly little tolerance toward other residue types at the K605 position (Figure 3C).

In an attempt to further increase the activity of Bad.F6Pkt on d-fructose, several combinations of advantageous single mutations were investigated. Unfortunately, none of the resulting double mutants exhibited a significant improvement in performance compared with the Bad.F6Pkt H548N variant (Figures S5 and S6).

### Combination of Mutations Q546E and N549D has a Synergistic Effect on d-Fructose Activity of Bad.F6Pkt

**Figure 4:**
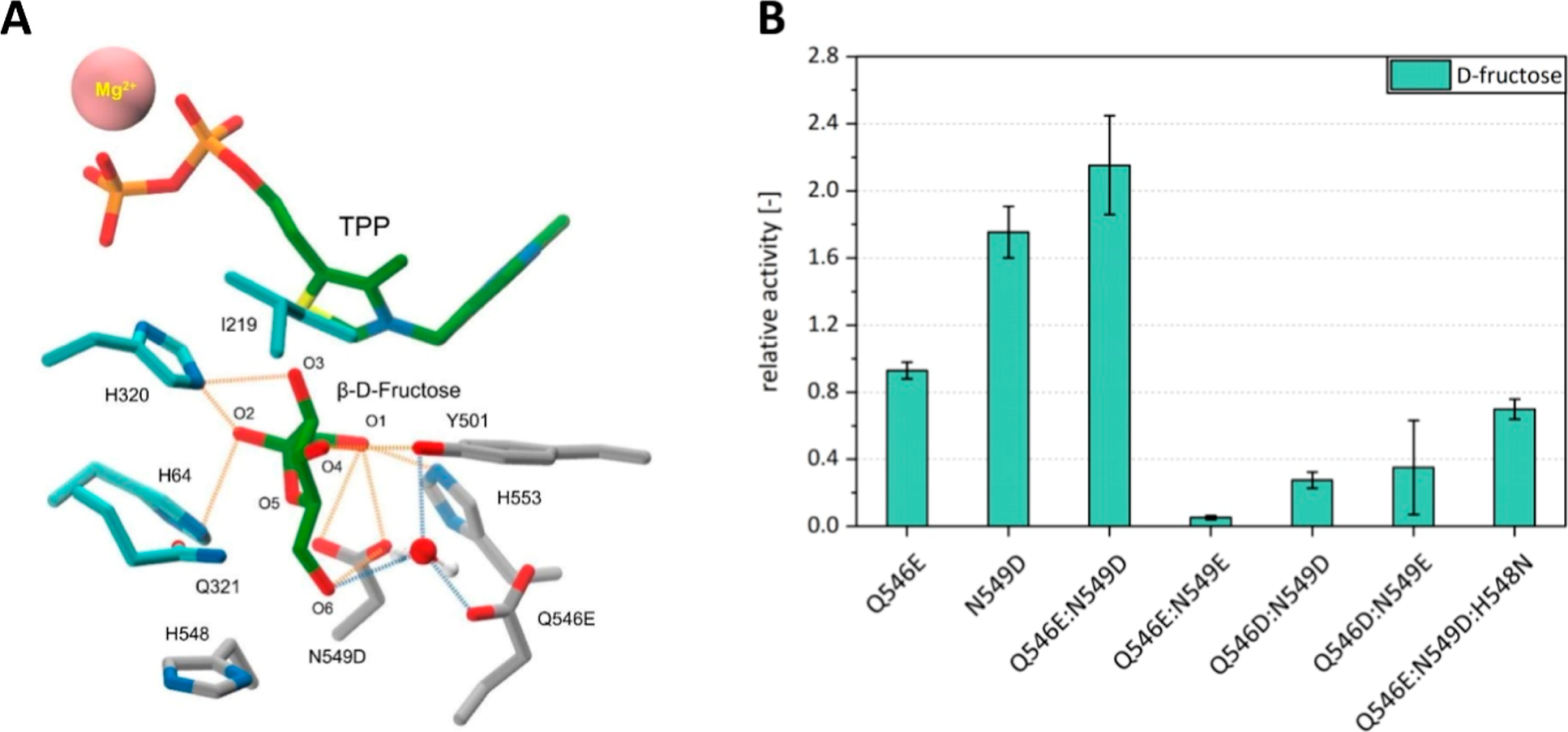
Mutations Q546E and N549D with synergistic effect on d-fructose activity of Bad.F6Pkt. (A) Model Bad.F6Pkt Q546E/N549D mutant complex with TPP and d-fructose. TPP and β-furanose fructose (FRU) ligands are shown in stick form with green colored carbons. The bound Mg^2+^ ion is depicted as a solid (pink) van der Waals sphere. Enzyme residue side-chain atoms are colored as described in the legend to Figure 2. Formal hydrogen bond vectors between FRU and the mutant enzyme atoms are shown in orange. Hydrogen bonding interactions of a hypothetical mediating water molecule, depicted in CPK representation, are colored blue. (B) Relative d-fructose-specific activity of Bad.F6Pkt variants carrying aspartate or glutamate in position Q546 and/or N549. Specific activity of purified enzymes was determined at pH 6.5, 37 °C in the presence of 200 mM d-fructose and 50 mM inorganic phosphate. Relative activity was determined as the ratio of mutant specific activity to that of the WT enzyme. Error bars indicate deviation of the mean (*n* ≥ 2).

Tittmann postulated that PKT binds the physiological substrate F6P in its cyclic form and then catalyzes *in situ* ring-opening to yield the linear form, which is eventually cleaved into an aldose phosphate and AcP. The ring-opening reaction was suggested to be assisted by the cognate substrate phosphate moiety, which may act as an acid–base catalyst via mediating solvent molecules.^34^ In aqueous solution, the abundancy of open-chain fructose is maximally only 2%, while the cyclic β-pyranose, β-furanose, α-pyranose, and α-furanose configurations amount to 65–72%, 21–25%, up to 3% and 5–6.5%, respectively.^35−38^ Due to the absence of a phosphate group in fructose, it seems likely that the capacity of the enzyme to catalyze ring-opening of the substrate is diminished, thereby limiting the overall reaction rate. Therefore, we aimed to engineer improved acid or base catalyzed fructose ring-opening within the Bad.F6Pkt active site. This was achieved by the conversion of N549 into a potential acid–base catalyst through mutation to aspartate and an attempt to incorporate a tight water-binding site capable of targeting the fructose O5 ring oxygen via single (Q546E) or double mutation (N549D/Q546E). A model complex of the Q546E/N549D mutant with TPP and the β-furanose cyclic fructose ring form (FRU) in the presence of a putative bound water molecule is illustrated in Figure 4A.

While no significant effect on fructose-specific activity was observed for the single mutation Q546E, an increase of 75% compared to the WT activity was found for mutation N549D. The combination of both mutations had a synergistic effect, as the double mutant Q546E/N549D exhibited up to 2.15-fold higher activity than the wild type enzyme (Figure 4B). Substrate saturation was not observed for this Bad.F6Pkt variant in the tested concentration range of up to 300 mM fructose (data not shown).

To investigate whether position N549 could be further exploited through the introduction of residues other than aspartate, a saturated mutagenesis library for this position was created and analyzed. With the exception of N549D, all mutations in this position resulted in a significant activity loss on d-fructose (Figure S7). With regard to the natural substrate F6P, a reduction in activity to 10% or below the WT level was observed for all N549 mutations.

In a further attempt to engineer the PKT toward improved fructose activity, we combined the mutations that were proposed to enhance substrate ring-opening with mutations in H548, the most promising target position identified from computational docking studies with the substrate in its linear configuration and corresponding library screening. Accordingly, a saturated mutant library in position H548 was introduced into the Bad.F6Pkt N549D and Bad.F6Pkt Q546E/N549D templates and screened in a 96-well plate format. In both cases, no Bad.F6Pkt variants with higher fructose-specific activity than the respective single mutant (N549D) or double mutant (Q546E/N549D) were identified (data not shown). The triple Bad.F6Pkt variant, which combines Q546E/N549D with the most effective single mutation in position 548 (H548N), had a 30% reduced fructose-specific activity compared to the WT (Figure 4B) and exhibited no substrate saturation (data not shown).

### Bad.F6Pkt H548N Variant Improves Performance of the Fructose-Based ATP Regeneration System

**Figure 5:**
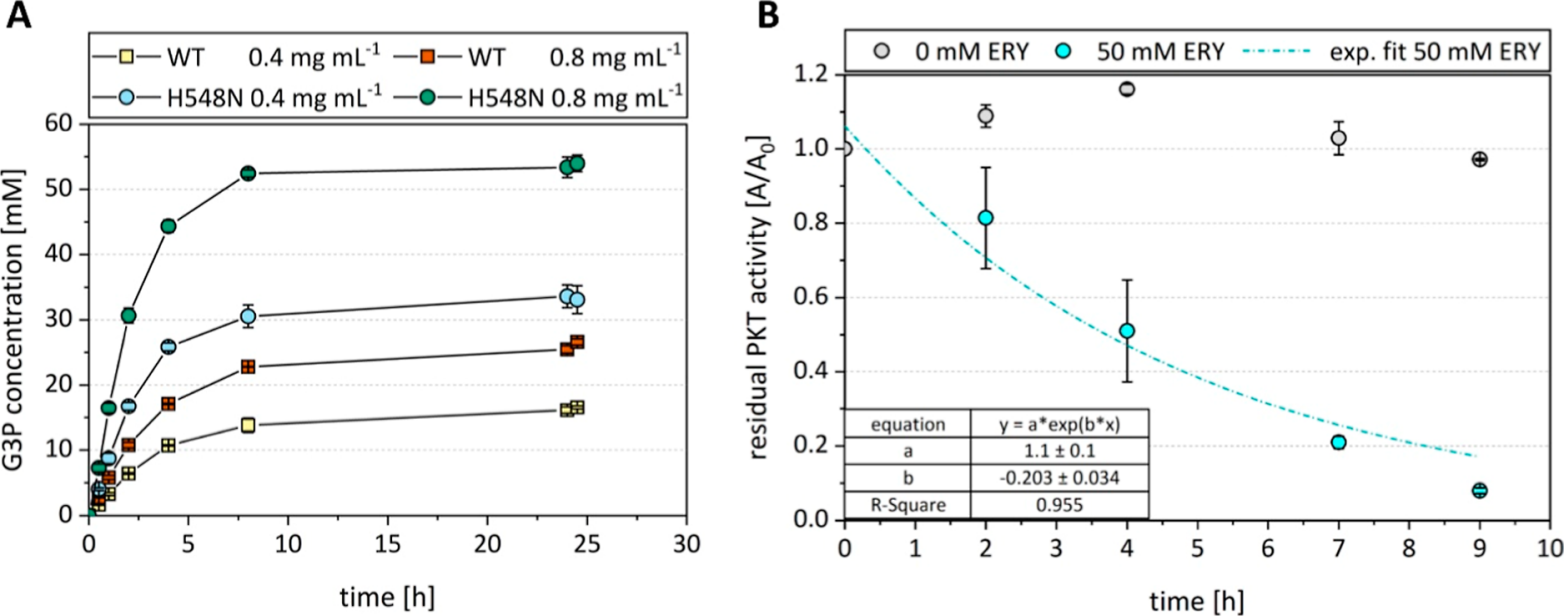
*In vitro* ATP regeneration from d-fructose by a PKT. (A) *In vitrosn*-glycerol 3-phosphate production with ATP regenerated from d-fructose. PKT activity was modulated by employing either WT Bad.F6Pkt or variant H548N at a concentration of 0.4 or 0.8 mg mL^–1^. Experiments were carried out at pH 7.0, 37 °C in the presence of 0.8 mM TPP, 4 mM MgCl_2_ with starting concentrations of 52 mM glycerol as limiting substrate, 100 mM sodium phosphate, 200 mM fructose, and 1 mM ADP. Data represent mean and deviation of biological duplicates. (B) Stability of Bad.F6Pkt H548N at synthesis-like conditions with and without the presence of d-erythrose (ERY). Incubation of 1 mg mL^–1^ purified PKT enzyme was carried out at 37 °C and pH 7.0 with 4 mM MgCl_2_, 50 mM glycerol, 75 mM inorganic phosphate, 200 mM fructose, and 1 mM ADP. The essential cofactor TPP was omitted from the reaction mix to prevent the PKT reaction. Samples were taken regularly and their activity was immediately determined by the hydroxamate assay. Data represent the mean and deviation of biological duplicates. Experimental data were fitted to the exponential equation using the exponential fit function of OriginPro 2021b (OriginLab Corporation, Northampton, US).

*In vitro* synthesis of *sn*-glycerol 3-phosphate (G3P) catalyzed by glycerol kinase from *Cellulomonas* sp. NT3060 was employed as model ATP-consuming reaction to characterize the cell-free, fructose-based ATP regeneration. The reaction system was completed by adding acetate kinase from *Geobacillus stearothermophilus* and either WT Bad.F6Pkt or the Bad.F6Pkt H548N mutant enzyme, which exhibits the highest activity on d-fructose. The reaction system was investigated using an initial glycerol concentration of 50 mM, 1 mM ADP, and excess d-fructose (200 mM). Complete conversion of glycerol to *sn*-glycerol 3-phosphate with a maximum productivity of 16.5 ± 0.8 mM h^–1^ was found using 0.8 mg mL^–1^ Bad.F6Pkt H548N, thus, demonstrating that ATP was regenerated from d-fructose as intended (Figure 5A). At a lower Bad.F6Pkt H548N concentration or when using the less active WT enzyme, the maximum productivity of G3P synthesis dropped to 8.7 ± 0.8 and 5.8 ± 0.3 mM h^–1^, respectively. Surprisingly, at all conditions tested here, the reactions stopped after approximately 8 h (Figure 5A) resulting in lower G3P yields when the productivity of the system was reduced. The analysis of substrates and (by)products by HPLC suggested that in the reactions with reduced productivity, fructose consumption stopped after 8 h (Figure S8). This led us to the assumption that the PKT is the limiting enzyme responsible for the low G3P yields.

The reason for the incomplete conversion of glycerol could be ascribed to the presence of d-erythrose, which is released in the PKT reaction. The C_4_ sugar does not inhibit the activity of Bad.F6Pkt H548N (not shown) but inactivates the enzyme when present at elevated concentrations (Figure 5B). Specifically, half-life of Bad.F6Pkt H548N was reduced from 144 to 3.4 h in the presence of 50 mM erythrose following first order kinetics with an inactivation constant of 0.203 h^–1^ (Table 2 and Figure 5B). From these results, we concluded that ATP regeneration from fructose is possible in principle and that the productivity of such a system can be improved by employing PKT enzymes with increased activity on fructose. However, for further increasing the performance of the system, the accumulation of inactivating erythrose concentrations must be prevented.

**Table 2:** Properties of Enzymes Used for *In Vitro* G3P Synthesis^e^

enzyme	Bad.F6Pkt	Bad.F6Pkt H548N	Ps.LrhI	Gs.AckA	Cs.GlpK
**organism**	*B. adolescentis*		*Pseudomonas stutzeri*	*Geobacillus stearothermophilus*	*Cellulomonas* sp. NT3060
**function**	phosphoketolase		erythrose isomerase	acetate kinase	glycerol kinase
**substrate**	**d****-fructose**		**d****-erythrose**	**AcP**	**glycerol**
*v*_max_ [U mg^–1^]	0.11 ± 0.03^a^	0.62 ± 0.08^a^	0.12 ± 0.01^b^	608 ± 57	8.0 ± 1.3
*K*_M_ [mM]	n.sat.	n.sat.	n.d.	2.3^c^	0.012^d^
*k*_cat_/*K*_M_ [M^–1^ s^–1^]	n.d.	n.d.	n.d.	(1.9 ± 0.2)*10^5^	(6.6 ± 0.8)*10^5^
**substrate**	**d****-erythrulose**			**ADP**	**ATP**
*v*_max_ [U mg^–1^]	0.30 ± 0.09	0.67 ± 0.12		608 ± 57	8.0 ± 1.3
*K*_M_ [mM]	36.2 ± 3.2	48.7 ± 0.4		0.8^c^	0.28^d^
*k*_cat_/*K*_M_ [M^–1^ s^–1^]	13.1 ± 4.5	21.3 ± 3.6		(5.5 ± 0.5)*10^5^	(2.8 ± 0.3)*10^4^
**substrate**	**glycolaldehyde**				
*v*_max_ [U mg^–1^]	0.41 ± 0.09^a^	0.53 ± 0.03^a^			
*K*_M_ [mM]	n.sat.	n.sat.			
*k*_cat_/*K*_M_ [M^–1^ s^–1^]	n.d.	n.d.			
**half-life [h]**	98 ± 8	144 ± 15	23 ± 2	47 ± 3	109 ± 1

a *v*_max_ values refer to the specific activity at a substrate concentration of 300 mM, since no saturation was observed under tested conditions.

b Activity measured in the presence of 10 mM d-erythrose and 4 mM MgCl_2_.

c Parameters estimated at pH 7.3, 30 °C.^40^

d Parameters estimated at pH 8.5, 30 °C, 1.5 mM MgCl_2_.^41^

e Half-life was determined by incubation at pH 7.0, 37 °C in the presence of 4 mM MgCl_2_, 1 mM ADP, 50 mM glycerol, and 75 mM inorganic phosphate at a total protein concentration of 1 mg mL^–1^. Unless otherwise stated, data were obtained in this work and represent mean and standard deviation of at least two experiments. n.sat.—no substrate saturation observed, n.d.—not determined.

### Extension of the Reaction System by d-Erythrose Isomerase Enables Higher ATP Yield

Figure 1 shows that complementation of the reaction system by d-erythrose isomerase does not only enable degradation of d-erythrose but also gives rise to higher ATP yields on fructose because both erythrulose and glycolaldehyde are potential substrates of PKT enzymes.^21,22^ The l-rhamnose isomerase from *Pseudomonas stutzeri* (Ps.LrhI) was previously found to have d-erythrose isomerase activity.^39^ We characterized the d-erythrose activity of the enzyme and its stability under synthesis-like conditions (Table 2). Although the specific activity of Ps.LrhI was rather low, its stability and activity were considered suitable for conducting proof-of-concept experiments. To fully characterize the reaction system, we measured the kinetic parameters of Bad.F6Pkt H548N and the WT enzyme on d-erythrulose and glycolaldehyde. We found that both enzymes had higher activities on the C_4_ and C_2_ substrates than on fructose. The Bad.F6Pkt H548N mutant had 2.2- and 1.3-fold higher activity on d-erythrulose and glycolaldehyde, respectively, than the WT enzyme (Table 2). Based on these results, we concluded that all required activities for implementing the reaction cascade depicted in Figure 1 could be provided by the enzymes listed in Table 2.

*In vitro* G3P synthesis by glycerol kinase, acetate kinase, and Bad.F6Pkt WT or variant H548N was tested in presence and absence of the erythrose isomerase Ps.LrhI. Here, glycerol was added in excess while fructose at a concentration of 25 mM was the limiting substrate in order to investigate whether a yield of more than 1 mol G3P per mol of C_6_ sugar is feasible.

**Figure 6:**
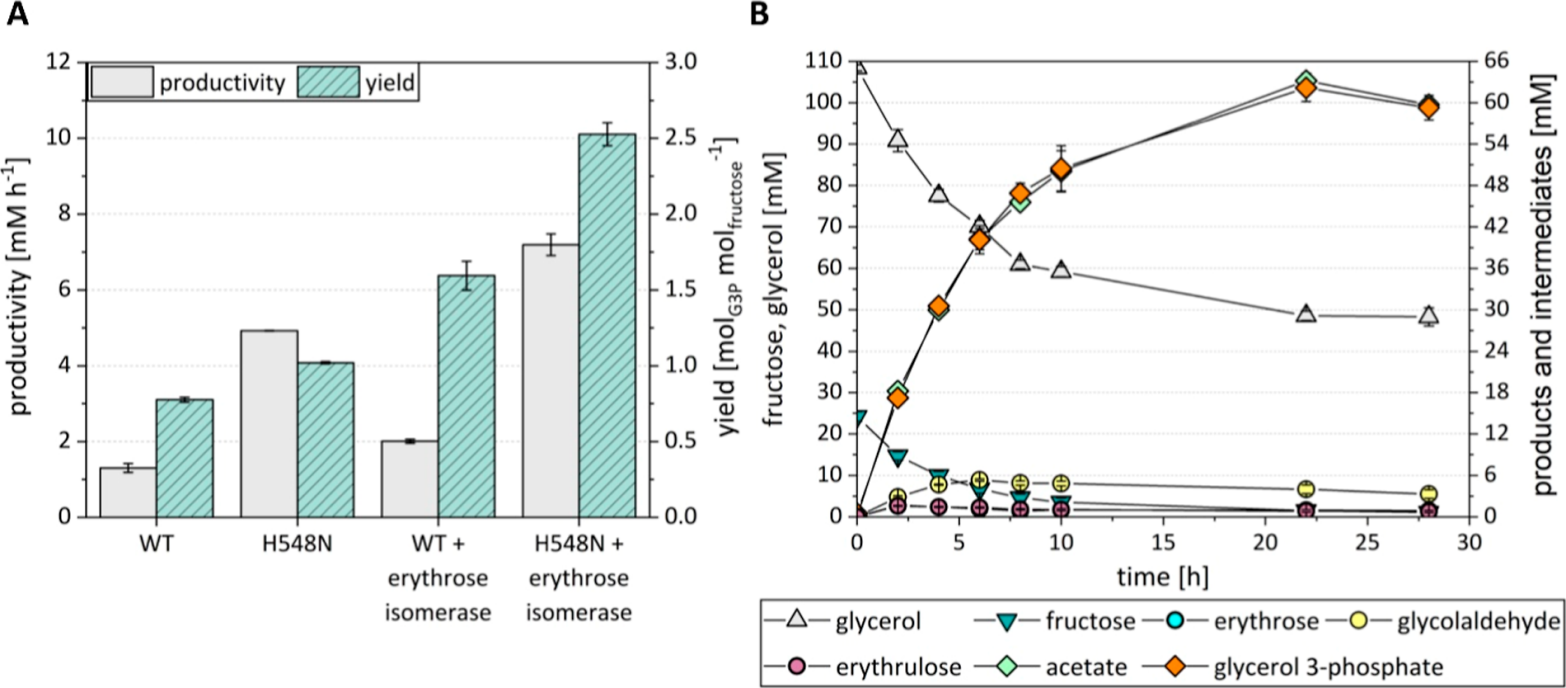
*In vitrosn*-glycerol 3-phosphate synthesis with 25 mM d-fructose as the limiting substrate for ATP regeneration. (A) Maximum productivity and yield of G3P synthesis using either Bad.F6Pkt H548N or the wild type enzyme, in the presence and absence of erythrose isomerase. Yield was calculated as the ratio of the maximum G3P and initial fructose concentrations. (B) Substrate consumption, accumulation of intermediates and (by-) products during *in vitro* G3P synthesis using Bad.F6Pkt H548N in the presence of erythrose isomerase. G3P synthesis was carried out at pH 7.0, 37 °C in the presence of 0.8 mM TPP, 4 mM MgCl_2_ with starting concentrations of 1 mM ADP, 110 mM sodium phosphate, and glycerol with 25 mM d-fructose as limiting substrate. PKT and erythrose isomerase were added at concentrations of 2.5 and 2 mg mL^–1^, respectively. Error bars indicate standard deviation of the mean (*n* ≥ 2).

The presence of erythrose isomerase increased the G3P yield from 0.78 ± 0.02 to 1.59 ± 0.1 mol_G3P_ mol_fructose_^–1^ using the WT PKT and from 1.02 ± 0.02 to 2.53 ± 0.07 mol_G3P_ mol_fructose_^–1^ when employing Bad.F6Pkt H548N (Figure 6A). At the same time, a 54% and 46% higher maximum productivity was observed, respectively. The highest yield and productivity were observed for the combination of Bad.F6Pkt H548N and erythrose isomerase, which enabled the synthesis of up to 62.18 ± 1.97 mM G3P. During the course of the reaction, up to 5.33 ± 0.15 mM glycolaldehyde accumulated (Figure 6B). At the end of the reaction, residual d-fructose concentration did not exceed 1 mM and the accumulation of 0.91 ± 0.09 mM erythrose, 0.78 ± 0.16 mM erythrulose, 3.31 ± 0.62 mM glycolaldehyde, and 59.7 ± 0.68 mM acetate (Figure 6B) was observed.

## Discussion

In this study, we proposed and successfully implemented a cell-free ATP regeneration system, which is based on the *in situ* production of the high-energy phosphoryl donor AcP from d-fructose and inorganic phosphate by an engineered PKT from *B. adolescentis*. It differs from previously proposed reaction systems for *in vitro* ATP regeneration by using a low-cost sugar substrate, preventing phosphate accumulation and rendering the system independent from aeration.

The conversion of the nonphosphorylated substrate fructose by WT PKTs was found to be inefficient, necessitating enzyme engineering to achieve sufficient reaction rates for the proposed PKT-based ATP regeneration system. Based on a molecular model of the template enzyme Bad.F6Pkt-TPP complex and substrate ligand docking studies, nine potential target positions for mutation were identified in the first contact shell. A single-mutation library screening at each target position revealed a Bad.F6Pkt mutation (H548N), which demonstrated a 5.6-fold increase in fructose activity relative to that of the WT enzyme. Our computational model of the TPP-fructose covalent adduct in the Bad.F6Pkt wild type and H548N mutant did not reveal the formation of a good quality hydrogen bond with the terminal hydroxyl group of the TPP-fructose adduct in either case as the side-chain in the mobile Q546-N549 (QN) loop appears to be orientated away from the C6 hydroxyl group (Figure 3B). In addition to H548, beneficial single variants were identified in positions Q321, S541, and K605. Again, none of these mutations show favorable nonbonded interactions with the terminal hydroxyl group of d-fructose as is apparent in Figure 3B. Indeed, the hydrogen bond interaction of Q321 with the fructose substrate is completely lost upon mutation to nonpolar leucine or valine residues. While restoration of the enzyme/substrate charge balance in the absence of a negatively charged substrate phosphate group can be assisted by mutation of K605 to valine or threonine, and the replacement of a potentially H548 charged species by asparagine, it is hypothesized that improvements in catalytic activity toward fructose in all the mutants chiefly derive from favorable induced preorganization of the electric field in the active-site center.

In support of this hypothesis, we note the presence of an aspartate (547) residue adjacent to H548 in the QN loop and spatially proximal to Q321, S541, and K605 residue positions (Figures 2 and 3B). Changes to the electrostatic polarity environment of D547 may result in shifts in its p*K*_a_ value relative to that in the WT enzyme. The unusually low experimentally determined p*K*_a_ value of 3.33 for aspartate 52 in hen egg white lysozyme has for example been ascribed to stabilization by nearby polar asparagine residues 44, 46, and 49.^42^ Nonconstructive induced electric field effects may thus account for why pairwise combinations of mutations at Q321, S541, and K605 positions did not result in increased activity compared to the best single mutant H548N. Similar observations (i.e., nonadditive effects of advantageous single mutations) were made by Yang et al.^22^ and Strafford et al.^43^ when engineering PKT or transketolase enzyme activity toward nonphosphorylated substrates.

It is noteworthy that H142 shields a glutamate residue (153) in the second substrate contact shell from the active site center (see Figure 3B). The origins of the improved catalytic activity on nonphosphorylated substrates reported in the H142N mutant by Yang et al.^22^ may be similar to those proposed here for H548N. MSA frequency profile analysis shows the presence of acidic aspartate or glutamate residues in 74% and 95% of PKT family sequences at positions 547 and 153, respectively (Figure S3A). Clearly, however, the precise mechanisms controlling substrate specificity of Bad.F6Pkt are not yet fully understood, and more elaborate computational, engineering, and screening methods will be required to achieve higher PKT activities on nonphosphorylated substrates by additive mutation at multiple sites.

We postulate that the intrinsically low fructose activity of PKT is due to a diminished capacity to catalyze sugar ring-opening in the absence of a substrate phosphate group. Following the introduction of mutations designed to facilitate acid or base catalysis for fructose ring-opening in the active site, a Bad.F6Pkt variant (Q546E/N549D) was identified, which exhibited a 2.15-fold higher activity than that of the WT. An aspartate residue in position 549, which is found in naturally occurring PKT sequences (Figure S3B), may afford improved hydrogen bonding with the O1 and O6 ring hydroxyl groups in addition to acting directly as an acid–base catalyst. On the other hand, the Q546E mutation is not observed in nature (Figure S3B), but might facilitate water-mediated targeting of the fructose O5 ring oxygen and indirect stabilization of the O6 sugar hydroxyl group (Figure 4A). Unfortunately, this double mutation that introduces significant additional negative charge into the active site was found to be incompatible with a mutation in H548. Consequently, the single Bad.F6pkt variant H548N is the most active on fructose to date.

The feasibility of ATP regeneration from d-fructose with a stoichiometry of 1 mol of ATP per mol of C_6_ ketose substrate was demonstrated by applying Bad.F6Pkt H548N together with an acetate kinase and a glycerol kinase for cell-free production of glycerol-3-phosphate from ADP and glycerol. Due to the low price of d-fructose, this reaction system would allow cell-free ATP production with substrate costs of only 0.3–0.9 € per mol ATP (calculated with a price of 1.42–4.72 € kg^–1^d-fructose,^44^ not considering costs for thiamine pyrophosphate, ADP and enzymes).

PKT was the limiting enzyme, and we observed time-dependent termination of the G3P synthesis reaction and incomplete substrate conversion when the productivity of the reaction system was too low. This phenomenon could be attributed to the accumulation of d-erythrose, which is a byproduct of the fructose-dependent PKT reaction, since Bad.F6Pkt H548N was found to be strongly inactivated in the presence of the C_4_ aldose. It was previously demonstrated that aldoses can inactivate enzymes through the process of glycation that is driven by their terminal aldehyde group.^45^ Together with our results, this indicates that ATP regeneration from fructose in a one-step PKT reaction is not feasible for most applications, as erythrose will inactivate enzymes of the ATP regeneration system (leading to suboptimal yields) and most likely damage other proteins. Therefore, avoiding the accumulation of high erythrose concentrations was mandatory for the ATP-regenerating enzyme cascade to work efficiently. Indeed, the addition of a d-erythrose isomerase to the reaction system enabled degradation of the C_4_ aldose to d-erythrulose and increased the ATP yield to 2.5 mol mol_fructose_^–1^, due to the fact that Bad.F6Pkt H548N is also able to convert d-erythrulose and subsequently glycolaldehyde to AcP.

Despite the utilization of high enzyme concentrations, the theoretical stoichiometry of three mol of ATP per mol of fructose was not attained. This observation was attributed to incomplete conversion of fructose and glycolaldehyde which emphasizes the need for improving enzyme activities on all nonphosphorylated substrates. Interestingly, Yang and colleagues were previously able to enhance the catalytic efficiency of PKT from *B. adolescentis* by a factor of 8.5 for glycolaldehyde and 3.6 for d-erythrulose through the incorporation of mutation H142N.^22^ The activities on these substrates were somewhat higher than those attained using the H458N variant that was developed in this study. However, the kinetic enzyme activity data and the time course of metabolite concentrations during operation of the full cascade indicated that the PKT activity on d-fructose clearly limited the overall productivity of the system. Since the activity of the Bad.F6Pkt H142N variant on fructose was only 23% of that of H458N, we did not use this enzyme for building the ATP-regenerating cascade.

In conclusion, the results of this study demonstrate the considerable potential of cell-free ATP regeneration from the inexpensive substrate fructose through the use of a PKT-based enzymatic cascade. The industrial relevance of this system will depend on further improvements of the PKT activities on the nonphosphorylated substrates and intermediates.

## Methods

### Reagents and Chemicals

Chemicals and solvents were purchased from Merck KGaA (Darmstadt, Germany) unless otherwise stated. Plasmid DNA isolation, gel DNA extraction, and PCR cleanup were performed using kits from New England Biolabs (Frankfurt am Main, Germany) according to the manufacturer’s instructions. Genomic DNAs were purchased from the German Collection of Microorganisms and Cell Cultures GmbH (DSMZ, Braunschweig, Germany). In their unavailability, synthetic genes were obtained from BioCat GmbH (Heidelberg, Germany) or Thermo Fisher Scientific Inc. (Waltham, MA, USA). DNA sanger sequencing was performed by Microsynth AG (Balgach, Switzerland) or Genewiz Germany GmbH (Leipzig, Germany).

### Plasmid Construction

Plasmids used in this study are listed in Table S5. Target genes were cloned into the pET-28a(+) expression vector (Novagen, Merck, Darmstadt, Germany), with the incorporation of a N-terminal hexa-His tag. The plasmid pET28a_Ps.lrhi was synthesized by BioCat GmbH (Heidelberg, Germany). Homologous recombination was used for all plasmid constructions carried out in this study. The pET-28a(+) plasmid backbone was amplified between the restriction sites NdeI and *Eco*RI by PCR, while the target genes were amplified with the introduction of backbone-homologues regions using the primers listed in Table S6. Remaining template DNA was digested by DpnI (NEB, 20,000 units/ml). After purification, the target PCR fragment and plasmid backbone were assembled by homologous recombination^46^ using the NEBuilder HiFi DNA Assembly Master Mix (New England Biolabs) according to the manufacturer’s instructions. The resulting products were transformed into chemically competent *E. coli* NEB5-α cells (New England Biolabs).

### Protein Production

N-terminally 6x-His-tagged enzymes were expressed by different *E. coli* host strains bearing the respective pET-28a(+) expression vector. The expression of acetate kinase was conducted in *E. coli* BL21 (DE3) cells (New England Biolabs), whereas all other enzymes were expressed in *E. coli* Rosetta(DE3) plysS (Merck KGaA) cells. Using the method described previously,^47^ enzymes were expressed at 25 °C for 20 h in 50 mL LB medium after induction with 1 mM isopropyl-β-d-thiogalactopyranoside. PKT production cultures were performed with autoinducing medium ZYM-5052,^48^ inoculated at an initial optical density at 600 nm (OD_600_) of 0.05, and incubated at 25 °C for 24 h.

### Protein Purification, Quantification, and Storage

Protein purification was performed as described previously^47^ with minor deviations. Briefly, the cell-free crude extract was incubated for 1 h at room temperature and 20 rpm rotation (Revolver, Labnet, Edison, NJ, USA) with 0.35 mL of Talon Cobalt affinity resin (Cytiva, Marlborough, USA). After two rounds of washing (10 mM KH_2_PO_4_, 300 mM NaCl, pH 7.5), His-tagged enzymes were eluted with 0.5 mL of elution buffer (10 mM KH_2_PO_4_, 300 mM NaCl, 500 mM imidazole, pH 7.0). Subsequently, the buffer was exchanged using a Amicon Ultra-0.5 centrifugal filter unit (pore size 10 kDa; Merck Millipore, USA). The protein fraction was recovered (1000 g, 4 °C, 2 min) in 0.4 mL of storage buffer (10 mM KH_2_PO_4_, 300 mM NaCl, pH 7.0) and stored at 4 °C until further use. Protein concentrations were quantified using the Bradford method (Roti-Quant^Ⓒ^, Carl Roth, Karlsruhe, Germany), with bovine serum albumin (0–100 μg mL^–1^) serving as the calibration standard.

### MSA Residue-Position Frequency Analyses

A partially phylogenetically diversity-filtered Hidden Markov model MSA was obtained from *hhblits* (^49^; version 3.3) searches of the (February 2023) Uniclust30 clustered sequence alignment database^50^ against the Bad.F6Pkt query sequence. Maximum accuracy (0.35) and minimum coverage (80%) settings were applied. The PKT MSA comprised a total of 1019 sequences, corresponding to an effective number of 739 (72.5%), calculated using the method of Morcos et al.^51^ with an identity cutoff of 80%. Deletion sites were not considered as an additional residue type.

Unweighted MSA position-dependent residue type frequencies and relative Shannon information entropy measures of residue variability (H_*X*_) were calculated using SEQUESTER.^52^ H_*X*_ is defined by
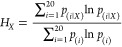
where *p*_(*i*|*X*)_ is the conditional probability of native residue type (*i*) occurrence at alignment position (*X*), and *p*_(*i*)_ is the normalized probability of residue type (*i*) occurrence at any position. Operationally, values for *p*_(*i*)_ here were taken from the documented global set for all natural proteins.^53^ Values of H_*X*_ vary continuously from zero, corresponding to a fully conserved residue position, to an upper limit of unity for a residue position showing no intrinsic residue type preference.

### Molecular Modeling of *B. adolescentis* Pkt Complexes with F6P and d-Fructose

An initial dimeric molecular model of *B. adolescentis* PKT (UniProtKB code A1A185), spanning the residue range V5–V806, was built automatically from the 2.1 Å homologue template X-ray structure (PDB entry 3AHI), bioassembly 1 coordinate set,^20^ of the *B. breve* Pkt H320A mutant (85% shared sequence identity; 100% sequence coverage) complex with acetyl TPP (AcTPP/HTL) using the SWISS-MODEL web server.^54^ Extended T2-W10 N-terminal sections were built into the two subunits by annealing of fragments of the same sequence obtained from an overlay of the Cryo-EM structure of the *B. longum* enzyme (PDB entry 6LXV(55)) on the 3AHI data set. The Bad.F6Pkt N-terminal methionine residues were built manually. Bound TPP cofactor atom coordinates were prepared by renaming of the HTL residue atoms following excision of the (C1′, O2′ C3′) atoms of the AcTPP acetyl group moiety. Mg^2+^ ion coordinate positions were similarly copied directly from the 3AHI data. Minor adjustments to the protein residue side-chain geometry and side-chain mutations were made using the COOT interactive molecular graphics and modeling package.^56^

The Bad.F6Pkt modeled complex with Mg-TPP was then energy minimized using the *ff99SB* Amber molecular mechanics force field for protein atoms^57^ in the presence of 1289 nonoverlapping (TIP3P) water molecules extracted from the 3AHI bioassembly coordinate set. GAFF force field parameters^58^ were used for TPP. The electrostatic model comprised a distance-dependent dielectric constant with ε = 4. Partial charges for TPP were abstracted from the set for the 4′-aminopyrimidine tautomeric form^59^ of TPP^2–^. The minimized Bad.F6Pkt model complex with Mg-TPP was then used as a template for the construction of the covalent ligand complex with the open-chain form of F6P. The highly conserved shape of the TPP binding pocket in *E. coli* transketolase^60^ permitted superposition docking of the TPP-F6P (T6F) covalent adduct from the 2r8p PDB coordinate data set at equivalent TPP heavy atom positions. The TPP-F6P covalent complex with Bad.F6Pkt was then subjected to a further round of energy minimization. Partial atomic charges for T6F were obtained by merging TPP^2–^ and terminal PO_4_^2–^ group charge sets with RESP charges^61^ from fitting to the quantum chemical electrostatic potential of a fully geometry-optimized structure of the d-fructose derivative of a thiazolium ring-containing TPP model analogue (Figure S2A). All Hartree–Fock quantum chemical calculations were performed with GAMESS^62^ using the 6-31G* basis set. A complete list of GAFF atom types and partial charges for atoms in the T6F CCD residue entry^63^ is provided in Table S1. The Pkt-TPP-d-fructose complex was prepared by excision of the terminal T6F phosphate group atoms.

A β-furanose ring template skeleton superposition docking protocol was implemented to construct a model complex of the cyclic form of d-fructose (FRU) with Bad.F6Pkt-TPP. A fructose bisphosphate (FBP) molecule, abstracted from the X-ray crystallographic complex with *Thermoproteus tenax* fructose 1,6-bisphosphate aldolase (PDB entry code 1W8S;^64^) was superposed at four structurally equivalent atom positions (P2, C5, O5, C3) on the bound T6F (P, C5, O5, C3) covalent adduct in the minimized complex with the enzyme. The β-furanose ring skeleton of the superposed FBP molecule overlapped closely with a β-d-ribofuranose 5-phosphate (RP5) ring skeleton from the complex with *E. coli* transketolase (PDB entry code 2R5N(60)), which was independently docked as a control by superposition over equivalent TPP atom positions. A HF/6-31G* geometry-optimized d-fructose ligand molecule was fitted to the superposition docked β-furanose ring template skeleton in the active site of the Bad.F6Pkt Q546E/N549D mutant. The double mutant-TPP noncovalent complex with FRU was then energy minimized as described above. A hypothetical water molecule was introduced at the centroid of the plane formed by the Q546E OE1, Y501 OH, and FRU O5 atoms, and a further round of energy minimization was carried out. GAFF force field atom types^58^ and RESP-fitted partial charges^61^ obtained from the optimized FRU geometry are tabulated in Table S2.

### Construction and Expression of *Bad.f6pkt* Libraries

Single site-directed mutagenesis of the *Bad.f6pkt* gene was performed by PCR according to the procedure described by Zheng and colleagues^65^ and the primers listed in the Supporting Information 4. Remaining template DNA was digested by DpnI restriction endonuclease before transformation of the PCR product into *E. coli* DH5α competent cells (New England Biolabs). Subsequent cultivation on LB agar plates supplemented with 50 μg mL^–1^ kanamycin yielded at least 300 colonies per transformation.

Double mutation libraries were created using the QuickChange Lightning Multi-Site-Directed Mutagenesis Kit (Agilent Technologies, Santa Clara, CA, US). Mutagenic primers were designed using the web-based QuickChange Primer Design Program by Agilent Technologies to allow a selected number of amino acids in each target position (as detailed in Tables S8–S11, primers listed in Supporting Information 4). The PCR for mutant strand synthesis, followed by DpnI digestion of remaining template DNA was performed according to the manufacturer’s instructions. The resulting PCR product was transformed into XL10-Gold ultracompetent cells provided by the commercial kit.

For each library, Plasmid DNA of a minimum of five randomly selected colonies was sequenced to evaluate the mutation efficiency and diversity of the library. The remaining colonies were scrapped of the agar plate and used to inoculate 20 mL LB medium supplemented with 50 μg mL^–1^ kanamycin in a 100 mL shake flask. After incubation overnight at 37 °C at 220 rpm agitation, plasmid DNA was isolated and transformed into *E. coli* Rosetta(DE3) plysS competent cells for protein expression.

The method for recombinant protein expression in 96 deep-well format was adapted from Irague et al.^66^ Media were supplemented with 50 μg mL^–1^ kanamycin and 35 μg mL^–1^ chloramphenicol. All cultivations were carried out at 25 °C, 850 rpm, and 85% humidity in an incubator shaker (Multitron, Infors AG, Switzerland). All 96-well plates were sealed with a Breath Easy membrane (Merck KGaA, Germany). Microplates (Falcon, Corning GmbH, Germany) containing 180 μL LB medium with 8% glycerol in each well were inoculated with *E. coli* clones picked from solid LB medium and incubated for 24 h. After storage at −80 °C, microplates were thawed, replicated, and used to inoculate the starter culture into 96-well microplates containing 180 μL of 2xYT medium (16 g L^–1^ tryptone, 10 g L^–1^ yeast extract, 5 g L^–1^ NaCl). Starter cultures were grown for 15 h, before 30 μL of each were transferred to 96-deep-well plates (Masterblock, Greiner Bio-One GmbH, Germany) filled with 600 μL autoinducing medium per well. This gene expression culture was incubated for 24 h prior to harvest by centrifugation at 2000 rpm and 4 °C for 20 min. After removal of 600 μL supernatant, cell pellets were resuspended in 100 μL of lysozyme solution (1 mg mL^–1^ in 10 mM Tris-HCl pH 8.0) and incubated at 30 °C, 250 rpm for 30 min prior to storage at −80 °C for at least 16 h.

### Library Screening in Terms of Activity on Nonphosphorylated Substrates

The cell pellets in deep-well plates were thawed for 1 h at room temperature, followed by addition of 100 μL of benzonase solution (15 U mL^–1^, 1 mM MgCl_2_) to each well and incubation at 30 °C, 250 rpm for 30 min. Storage buffer (10 mM KH_2_PO_4_, 300 mM NaCl, pH 7.0) was added in aliquots of 180 μL per well before centrifugation at 2000 rpm and 4 °C for 20 min. The obtained enzymatic extracts were stored on ice until they were assessed in terms of PKT activity on d-fructose as described below.

The number of clones analyzed for each library was determined to be three times higher than the total number of possible variants to ensure a library coverage of ≥95%.^67^ As an internal reference, at least three WT colonies were included on each plate.

### Enzymatic Assays

Enzyme activities were determined by photometric methods using the Infinite M200 PRO plate reader (Tecan AG, Switzerland), I-control software (version 3.8.2.0, Tecan AG), and 96-well flat bottomed microplates (Thermo Fisher Scientific Inc.). One unit of enzyme activity (U) is defined as synthesis of 1 micro mole of product per minute.

The enzymatic activity of PKT was determined by detecting the product AcP via the hydroxamate assay.^28,29^ This method is based on the spectrophotometric quantification of an iron–acetyl–hydroxamate complex formed by the derivatization of AcP. The reaction mixture contained 100 mM MES buffer pH 6.5, 50 mM KH_2_PO_4_, 3.8 mM MgCl_2_, 0.8 mM TPP, 1.4 mM l-cysteine, 200 mM d-fructose, and 20 μL purified protein solution in a total reaction volume of 80 μL. To measure PKT activity in cell-free crude extracts, 17 mM NaF and 6 mM iodoacetate were additionally present. The reaction mixture was incubated at 37 °C for 30 min, before 60 μL of hydroxylamine solution (2 M, pH 6.5) was added to stop the enzymatic reaction. After incubation for 10 min at room temperature, 40 μL of trichloroacetic acid (15% w/v), 40 μL of HCl (4 M), and 40 μL of FeCl_3_*6 H_2_O solution (5% w/v in 0.1 M HCl) were added. Absorbance was measured immediately at 505 nm. Control reactions with the absence of substrate, protein or both were included. Calibration was performed with freshly prepared lithium AcP standard solutions (0–14 mM).

In order to estimate kinetic parameters of PKT for different substrates, specific activity was measured at sugar substrate concentrations ranging from 0 to 100 mM (F6P, d-erythrulose) or 0–300 mM (d-fructose, glycolaldehyde). The Michaelis constant (*K*_M_) and maximum reaction velocity (*v*_max_) were determined by fitting experimental data to the Michaelis–Menten or substrate inhibition model using the Curve fitting tool of MatLab software (version R2023b, Mathworks Inc., Natick, MA, USA).

d-erythrose isomerase activity was determined by the quantification of d-erythrulose production in a coupled assay utilizing d-erythrulose reductase from *Gallus gallus* (Gg.DER). The method was adapted from the procedure described by Morii and colleagues.^68^ The reductase enzyme was expressed in shake flasks and purified as described above, prior to storage at −20 °C in a solution containing 8 mM KH_2_PO_4_, 237 mM NaCl, 10% glycerol, and 1 mM dithiothreitol. The reaction mixture consisted of 100 mM HEPES buffer pH 7.0, 4 mM MgCl_2_, 0.2 mM NADH (dissolved in 10 mM NaOH), and 8 μg mL^–1^ Gg.DER, along with appropriate quantities of the purified isomerase enzyme, in a total volume of 250 μL. The addition of 10 mM d-erythrose initiated the enzymatic reaction, which was then monitored continuously at 37 °C by detecting NADH at 340 nm. The stoichiometry of NADH consumption by the reductase coupled to d-erythrulose production by the d-erythrose isomerase is 1:1.

The activity of acetate kinase was quantified by the detection of ATP through a coupled assay with hexokinase (Roche Diagnostics GmbH, Mannheim, Germany) and glucose 6-phosphate dehydrogenase (Sigma-Aldrich, Saint Louis, MO, USA) based on the procedure described by Aceti and Ferry.^69^ In a total volume of 250 μL, the reaction mixture contained 50 mM HEPES buffer pH 7.0, 10 mM MgCl_2_, 5 mM ADP, 1 mM dithiothreitol, 10 mM glucose, 1 mM NADP (dissolved in 10 mM NaOH), 20 U mL^–1^ hexokinase, 10 U mL^–1^ glucose 6-phosphate dehydrogenase, and appropriate quantities of the purified enzyme. The enzymatic reaction was initiated by addition of 20 mM AcP, performed at 37 °C and followed continuously by the detection of NADPH at 340 nm.

Activity of glycerol kinase was determined by quantification of *sn*-glycerol 3-phosphate production using the method described below. The reaction mixture additionally contained 5 mM ATP and the enzymatic reaction was started by the addition of 50 mM glycerol.

### *In VitroSn*-Glycerol 3-Phosphate (G3P) Synthesis

Solutions of purified enzymes were prepared, quantified, and stored as described above prior to their application for *in vitro* G3P synthesis. The enzymatic synthesis of *sn*-glycerol 3-phosphate was carried out in a 2 mL tube with a total reaction volume of 1 mL. Unless otherwise stated, the reaction mixture comprised 200 mM HEPES buffer pH 7.0, 1 mM ATP, 0.8 mM TPP, 4 mM MgCl_2_, 100 mM sodium phosphate pH 7.0 and glycerol, 5 μg mL^–1^ Gs.AckA, and 0.3 mg mL^–1^ Cs.GlpK. Bad.F6Pkt (variants) and erythrose isomerase were applied as indicated. The reaction mixture was preincubated at 37 °C for 5 min before the reaction was initiated by addition of d-fructose at a concentration of 25 mM. Incubation was carried out at 37 °C for ∼30 h, while 50 μL samples were taken regularly from the reaction mixture, diluted 1:4 in 0.1 M HCl and placed on ice to stop the enzymatic reaction. Part of this sample solution was used in-time for offline monitoring of G3P production, while the remaining part was stored at −20 °C until further use.

### Photometric Quantification of G3P

G3P was quantified by a coupled enzymatic assay using *sn*-glycerol 3-phosphate oxidase (G3Pox) and peroxidase. The G3Pox apoenzyme (Creative Enzymes, USA) was reactivated by incubation for 2 h at 4 °C in 50 mM HEPES buffer pH 7.5 supplemented with 0.25 mM FAD. At these conditions, the enzyme was stored up to 3 weeks. Samples were diluted appropriately in 0.1 M HEPES buffer at pH 7.5. Subsequently, 50 μL diluted samples were added to the assay mixture containing 50 mM HEPES buffer pH 7.5, 10 mM MgCl_2_, 7 mM sodium 3,5-dichloro-2-hydroxybenzenesulfonate pH 7.5, 2 mM 4-amino antipyrine, 2 U mL^–1^ G3Pox, and 50 U mL^–1^ horseradish peroxidase (Merck KGaA, Germany). The assay mixture with a total volume of 250 μL was incubated at 37 °C while the absorbance was read at 520 nm every 20 s until a constant signal was observed. G3P concentration was determined based on the absorption difference of the sample reaction and a control reaction without G3Pox.

### Quantification of Substrates, Intermediates, and Byproducts

High-performance liquid chromatography (UltiMate3000, Thermo Fisher Scientific, USA) was used for the quantification of fructose, erythrose, erythrulose, threose, glycolaldehyde, glycerol, and acetate. The system was equipped with a Rezex ROA-Organic Acid H^+^ (8%) column (Phenomenex), a DAD-detector (DAD-3000(RS), Thermo Fisher Scientific, USA), and a RI-detector (RefractoMax 520, ERC, Germany). Samples were appropriately diluted in ddH_2_O and processed through a 0.2 μm PTFE filter before 20 μL was injected to the column. Elution of the analytes was carried out with 0.5 mM H_2_SO_4_ at a flow rate of 0.5 mL min^–1^ and a temperature of 65 °C. The temperature of the autosampler was set to 6 °C.

## Supporting Information Available

Additional computational structure and sequence analysis results, including MSA residue frequency profiles, partial charges, and GAFF atom types; additional experimental results, including the characterization of PKT candidate enzymes, screening of Bad.F6Pkt variants, and fructose consumption during *in vitro* glycerol 3-phosphate synthesis; and additional material and methods, including plasmids used in this study, expression conditions, and library design (PDF)
Primers used for construction of *Bad.f6pkt* libraries and individual incorporation of mutations into the *Bad.f6pkt* gene (XLSX)
