# Supplementary material for: Cell-Free Reaction System for ATP Regeneration from d-Fructose
Source: ACS Synth Biol. 2025 Mar 27;14(4):1250–63. doi: 10.1021/acssynbio.4c00877 (PMC12012885; doi:10.1021/acssynbio.4c00877)
Supplement: Supplementary file 1 — sb4c00877_si_001.pdf [file sb4c00877_si_001.pdf]

Supporting Information (1 – 3)

Cell-free reaction system for ATP regeneration from D-fructose

Franziska Kraußer[1], Kenny Rabe[1], Christopher M Topham[2], Julian Voland[1♦], Laura Lilienthal[1], Jan-Ole Kundoch[3], Daniel Ohde[3], Andreas Liese[3], Thomas Walther[1*]

[1]Chair of Bioprocess Engineering, Institute of Natural Materials Technology, TU Dresden, Bergstraße 120, 01062 Dresden, Germany

[2] Molecular Forces Consulting, 24 Avenue Jacques Besse, 81500 Lavaur, France

[3] Institute of Technical Biocatalysis, Hamburg University of Technology, Denickestr. 15, 21073 Hamburg, Germany

***Email:** thomas_walther@tu-dresden.de

♦ J.V. Deceased in April 2024

Supporting Information 1: Additional computational structure and sequence analysis results

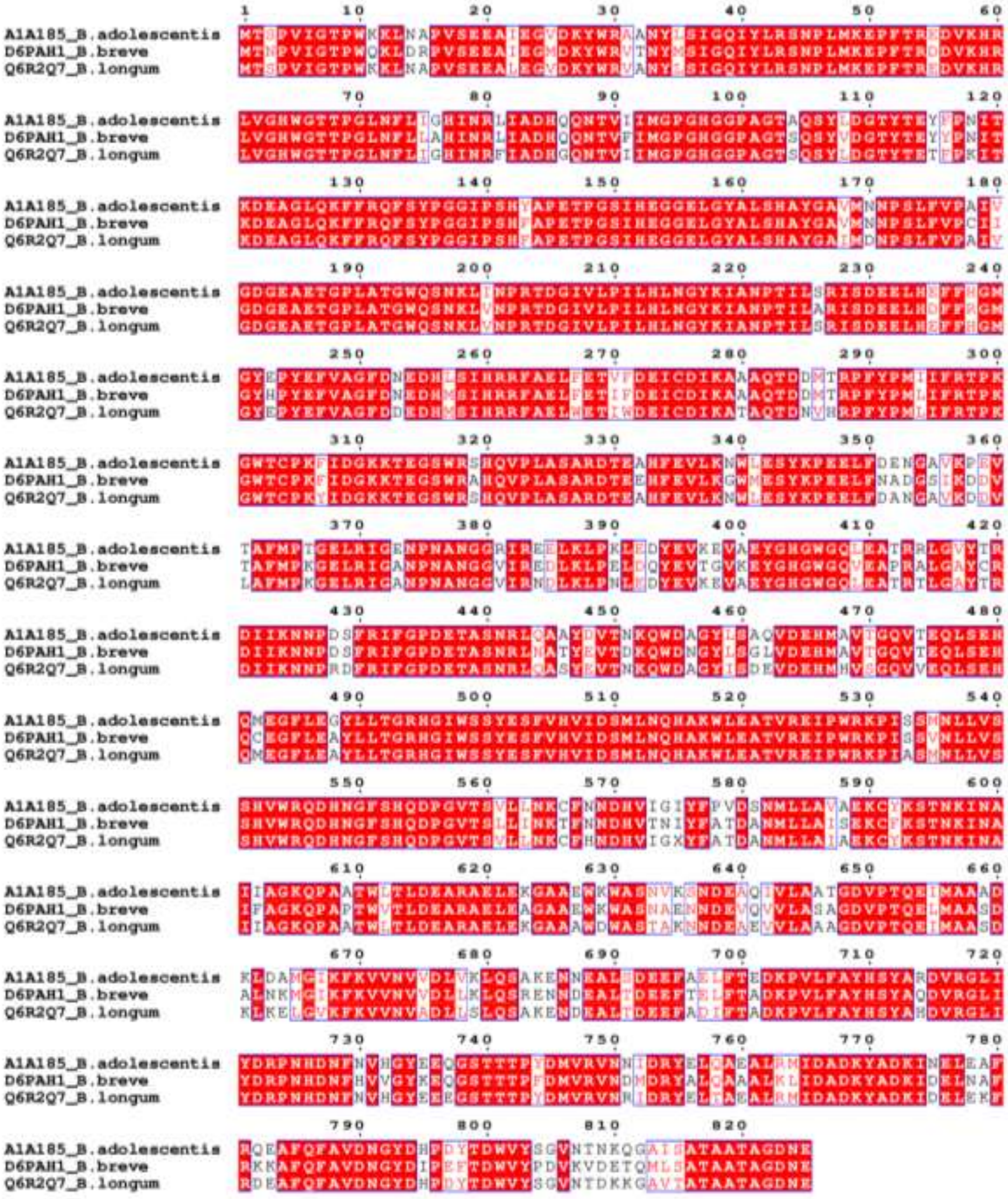

Figure S1 Sequence alignment of the phosphoketolases from *B. adolescentis, B. breve* and *B. longum*. Clustal Omega program[1] via the EMBL-EBI Job Dispatcher sequence analysis tools framework (https://www.ebi.ac.uk/jdispatcher, [2]) was used to perform the sequence alignment. The figure was created with the ESPript 3.0 program (https://espript.ibcp.fr, [3]) using a global similarity score of 0.7. The residues in each column are coloured according to the Risler score, where white letters in red boxes indicate strict identity. Similar residues between phosphoketolases are shown as red letters within blue frames.

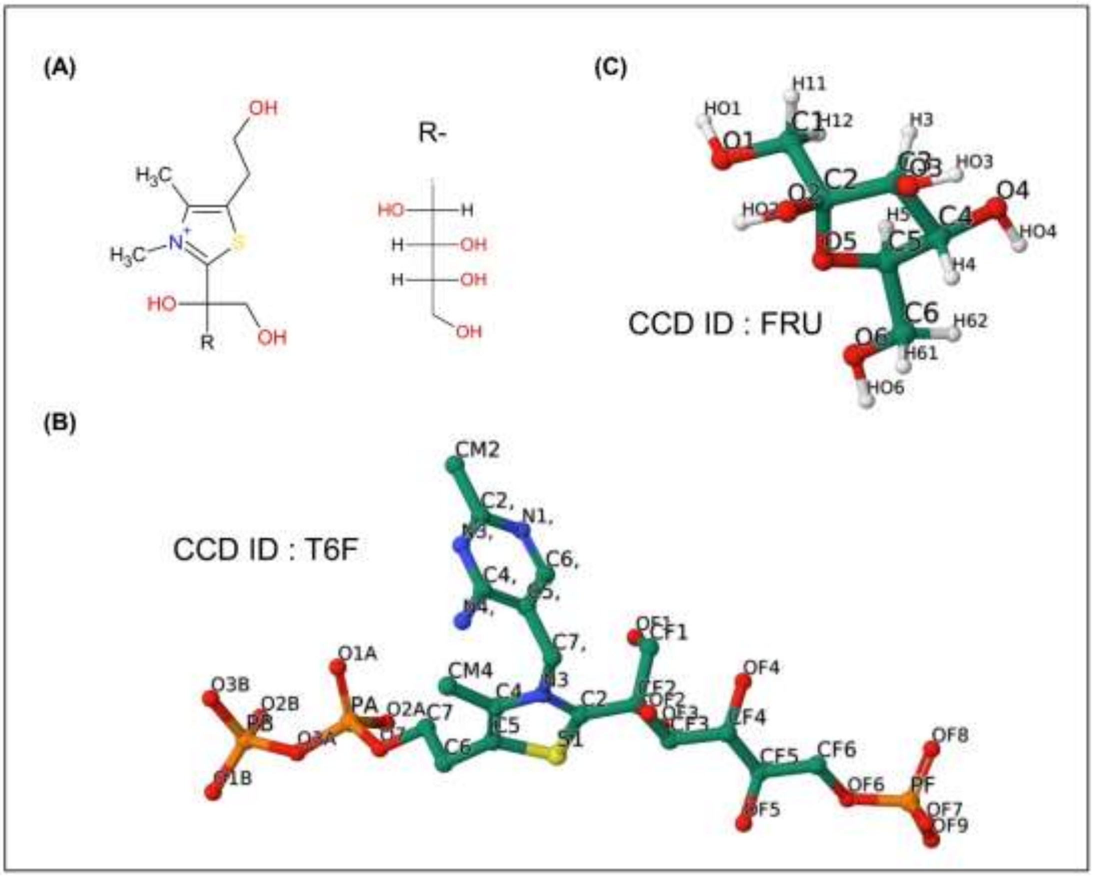

Figure S2 Model compound chemical and ligand structures used in partial atom charge fitting. (A) D-Fructose donor substrate adduct derivative of 2-(1,2-dihydroxyethyl)-5-(2-hydroxyethyl)-3,4-dimethyl-1,3-thiazol-3-ium TPP analogue fragment. **(B)** Chemical Components Dictionary (CCD) residue entry [4] atom names of TPP-fructose-6-P (T6F) covalent adduct and **(C)** cyclic β-furanose form of D-fructose (FRU).

Table S1 Partial charges and GAFF atom types for TPP-fructose-6-P (T6F) covalent adduct. Heavy atom names are indicated in Figure S2-B.

Atom Name	Partial Charge	GAFF Atom Type		Atom Name	Partial Charge	GAFF Atom Type
PA	1.500000	p5		H61	0.120823	hc
O1A	-0.820000	o		H62	0.120823	hc
O2A	-0.820000	o		H71	0.197491	h1
O3A	-0.740000	os		H72	0.197491	h1
PB	1.100000	p5		HOF2	0.462900	ho
O1B	-0.900000	o		HF11	0.105700	h1
O2B	-0.900000	o		HF12	0.105700	h1
O3B	-0.900000	o		HOF1	0.477900	ho
O7	-0.620000	os		HF3	0.121400	h1
C7	-0.294982	c3		HOF3	0.445300	ho
C6	0.321007	c3		HF4	0.133300	h1
C5	-0.459048	cc		HOF4	0.435000	ho
S1	0.466983	ss		HF5	0.132200	h1
C2	-0.343096	cc		HOF5	0.498000	ho
N3	0.504082	na		HF61	0.062600	h1
C4	0.018769	cd		HF62	0.062600	h1
CM4	-0.167640	c3		HM41	0.077470	hc
CF2	0.033500	c3		HM42	0.077470	hc
OF2	-0.616500	oh		HM43	0.077470	hc
CF1	0.067900	c3		H7,1	0.074473	h1
OF1	-0.665800	oh		H7,2	0.074473	h1
CF3	0.167900	c3		H4,1	0.401607	hn
OF3	-0.608200	oh		H4,2	0.467959	hn
CF4	0.184100	c3		HM21	0.113185	hc
OF4	-0.653800	oh		HM22	0.113185	hc
CF5	0.060200	c3		HM23	0.113185	hc
OF5	-0.718600	oh		H6,	0.084579	h4
CF6	0.120100	c3				
OF6	-0.740000	os				
PF	1.100000	p5				
OF8	-0.900000	o				
OF7	-0.900000	o				
OF9	-0.900000	o				
N1,	-0.872533	nb				
C2,	0.907369	ca				
CM2	-0.377400	c3				
N3,	-0.854045	nb				
C4,	0.934389	ca				
N4,	-1.008078	nh				
C5,	-0.536339	ca				
C6,	0.512937	ca				
C7,	-0.037442	c3				

Table S2 Partial charges and GAFF atom types for cyclic β-furanose form of D-fructose (FRU). Atom names are given in Figure S2-C.

Atom Name	Partial Charge	GAFF Atom Type
C1	0.1738	c3
C2	0.3600	c3
C3	0.0657	c3
C4	0.2154	c3
C5	0.0624	c3
C6	0.1751	c3
O1	-0.6711	oh
O2	-0.6037	oh
O3	-0.6477	oh
O4	-0.6934	oh
O5	-0.3690	os
O6	-0.6729	oh
H11	0.0339	h1
H12	0.0339	h1
H3	0.0955	h1
H4	0.0589	h1
H5	0.1152	h1
H61	0.0233	h1
H62	0.0233	h1
HO1	0.4411	ho
HO2	0.4472	ho
HO3	0.4608	ho
HO4	0.4386	ho
HO6	0.4337	ho

Table S3 Bad.F6Pkt residue positions in first contact shell of model complex with TPP fructose-6-P adduct. Upper inter-atomic contact distance limit set as 5 Å. Proximal substrate carbon positions are indicated in parentheses. *Residue positions mutated in this study. (‡) Relative Shannon entropy (H_x) values calculated from PKT family multiple sequence alignment as described in Methods.

Residue	Domain	Structural Element	Active-Site Location and Putative Mechanistic Role	MSA Relative Entropy (H_X)‡
HIS 64	PP	Coil	Inorganic Pi binding site; Substrate binding channel (C3/C4); Putative proton acceptor in deprotonation of C3 hydroxyl in C3-C4 bond cleavage.	0.028
HIS 97	PP	Helix	H-bond to expelled water molecule	0.016
HIS 142*	PP	Coil	Lines substrate binding channel (C1); Putative H-bond to expelled water; Potential proton donor in dehydration	0.046
ILE 219*	PP	Coil	Lines substrate binding channel (C3)	0.149
HIS 320	PP	Coil	Inorganic Pi binding site; Lines substrate binding channel (C3); Putative proton acceptor in deprotonation of C3 hydroxyl group	0.018
GLN 321*	PP	Coil	Inorganic Pi binding site; Substrate binding channel (C5)	0.284
GLU 437*	PYR	Coil	Closest contact with N3 TPP aminopyrimidine ring atom; Side-chain carboxylate / carboxylic acid group shielded from solvent by TYR 501	0.058
SER 440*	PYR	Coil	Substrate binding channel (C6)	0.035
ARG 442*	PYR	+ve ϕ	F6P phosphate group recognition	0.406
TYR 501	PYR	Coil	Inorganic Pi binding site; Lines substrate binding channel (C5/C6)	0.029
SER 541*	PYR	+ve ϕ	Lines substrate binding channel (C6)	0.137
GLN 546*	PYR	Coil	QN mobile loop in substrate binding channel (C6)	0.156
ASP 547	PYR	Coil	Withdrawn shielded negative charge centre in mobile QN loop	0.368
HIS 548*	PYR	Coil	QN mobile loop in substrate binding channel (C6)	0.159
ASN 549*	PYR	Coil	Inorganic Pi binding site; QN mobile loop in substrate binding channel (C4); Potential role in 5-membered ring opening	0.033
HIS 553	PYR	Helix	Lines substrate binding channel (C2); Potential proton donor in dehydration	0.015
LYS 605*	PYR	Coil	F6P phosphate group recognition	0.052

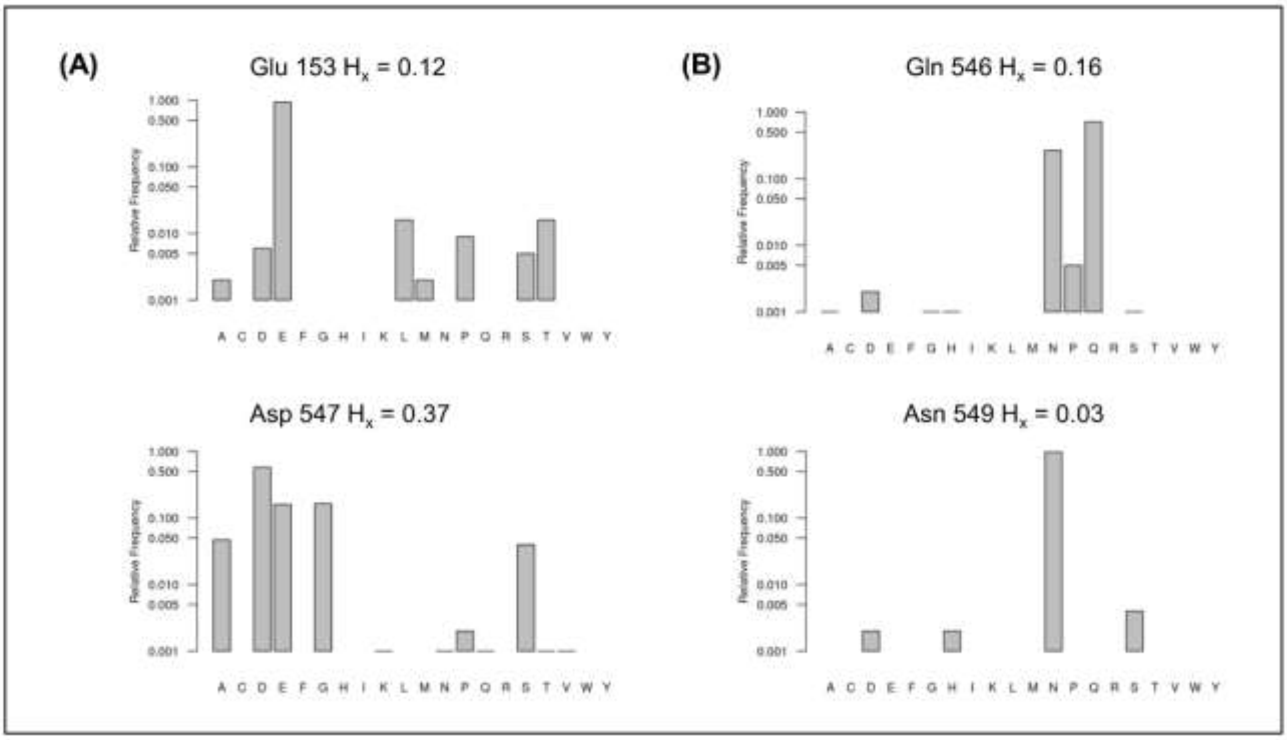

Figure S3 MSA residue frequency profiles. (A) Solvent shielded acidic residue positions in active-site region of Bad.F6Pkt. **(B)** Residue positions in Q546E:N549D double mutant.

Supporting Information 2: Additional experimental results

Table S4 Properties of PKT template enzyme candidates. Optimal expression conditions were established by a first round of screening. CV – culture volume, n.sat. – no substrate saturation observed. Data represent the mean and deviation of at least two biological replicates.

Abbreviation	Bad.F6Pkt	Bb.Pkt	Bb.Xfp	Ca.Xfp
**UniProtKB code**	A1A185	D4BMR5	D6PAH1	Q97JE3
**Source organism**	*Bifidobacterium adolescentis* ATCC 15703	*Bifidobacterium breve* DSM 20213	*Bifidobacterium breve* strain 203	*Clostridium acetobutylicum* ATCC 824
**D-fructose 6-phosphate**				
K_M [mM]	10.52 (± 0.26)	7.57 (± 0.62)	8.44 (± 0.30)	13.75 (± 1.25)
v_{max} [U mg^{-1}]	7.37 (± 0.29)	2.94 (± 0.43)	1.97 (± 0.20)	0.73 (± 0.12)
**D-fructose**				
K_M [mM]	n.sat.	n.sat.	n.sat.	n.sat.
V_{300mM} [U mg^{-1}]*	0.112 (± 0.029)	0.029 (± 0.005)	0.033 (± 0.002)	0.018 (± 0.002)
**Protein yield [$\mu g_{Prot.}$ mL_{CV}^{-1}]**	53 (± 8)	63 (± 2)	38 (± 6)	6 (± 1)

* Since substrate saturation was not observed within the concentration range tested, the specific activity in the presence of 300 mM D-fructose (v_{300mM}) is given here instead of v_{max}.

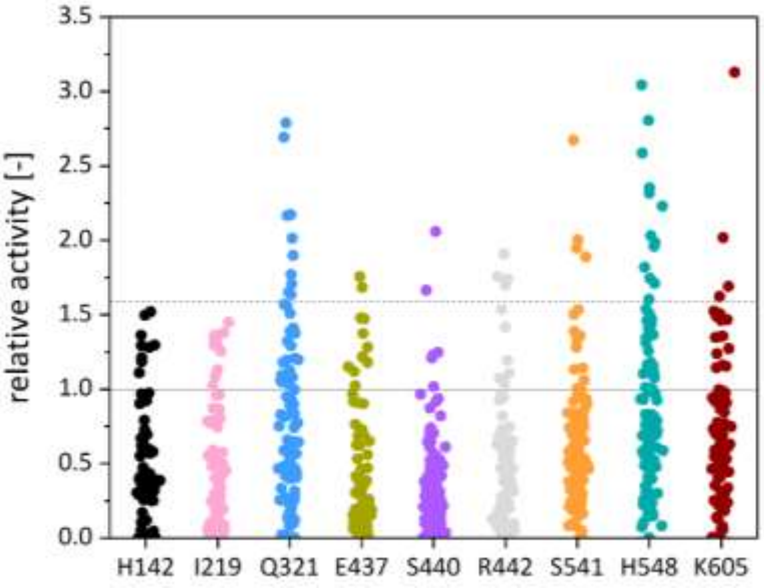

Figure S4 Screening of single mutation libraries: relative D-fructose activity of Bad.F6Pkt variants. Expression was carried out in 96 deep-well plates using auto-induction medium. Crude extract activity was measured at pH 6.5, 37 °C in the presence of 300 mM D-fructose. Relative activity was determined as the ratio of the specific activity of the mutant and wild-type control included on each plate. Solid grey line refers to the mean wild type activity, while the dashed line marks the hit threshold. The hit threshold was set to an activity of three standard deviations higher than the mean of the wild type activity.

Combining advantageous single mutations does not yield a further activity increase

In order to further enhance Bad.F6Pkt activity on fructose, we first tested whether the beneficial effect of mutations in each position was additive. We constructed enzymes containing pairwise combinations of the best single mutations Q321L, S541N, H548N, K605T (resulting in six variants) and assayed their activities of the purified proteins. However, none of the double mutants exhibited increased fructose activity (Figure S5).

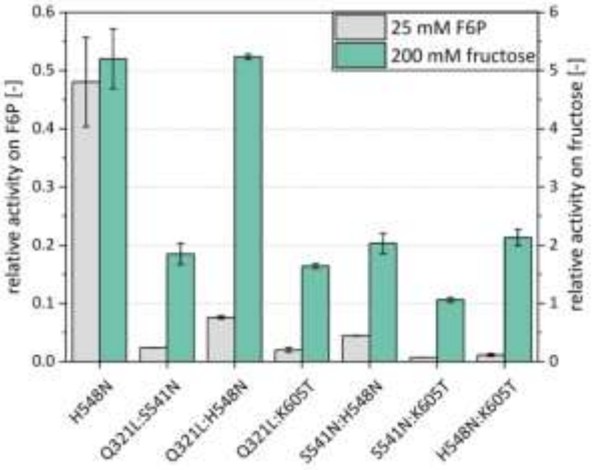

Figure S5 Combination of advantageous Bad.F6Pkt single mutations: relative D-fructose and fructose 6-phosphate (F6P) activity. Protein expression was carried out in shake flasks at 25 °C for 24 h using auto-induction medium. Activity of purified enzyme variants was measured at 37 °C and pH 6.5 in the presence of 50 mM inorganic phosphate. Relative activity was determined as the ratio of the specific activities of the mutant and wild-type enzymes**.** Data are presented as mean and standard deviation (n ≥ 2).

Subsequently, more variability was permitted in the four target positions to allow for potentially positive interactions of amino acid residues which individually did not give rise to the highest activity increase. Given that single mutations in position H548 gave rise to the highest activity increase on fructose, and because increased activity was observed for a comparatively large number of amino acid residues with diverse physical properties, mutations at this site were incorporated into all focused, double mutation libraries. In addition to mutations in H548, each library contained variations in one of the other three beneficial target positions – either Q321, S541 or K605. In addition to K605 and H548, the side-chain of the positively charged R442 residue also appears to interact with the F6P phosphate moiety (see Figure 2). Modulating R442 to compensate for the loss of the doubly negatively charged phosphate group in the target substrate fructose might restore the enzyme/substrate charge balance in Bad.F6Pkt mutated at position H548 or K605. Therefore, we also combined mutations at position H548 with variations in R442, despite the fact that single mutations in this position did not result in an increase in fructose activity.

All focused libraries comprised residues with disparate physical properties in both mutation sites, as detailed in Table S8 – S11. Briefly, in each position, a positively charged amino acid, one with a negatively charged side-chain, as well as at least one hydrophilic, hydrophobic and aromatic residue were allowed. Since the side-chain in position Q321 is implicated in the binding of the negatively charged inorganic phosphate [5], we did not allow an acidic residue in this position to avoid deleterious effects on P_i affinity. In total, we constructed four focused libraries containing 188 possible variants which were expressed in deep well plates and characterized in microtiter plate assays. Despite the identification of multiple variants exhibiting higher-than-wild-type activity on D-fructose, none of these variants demonstrated a significant improvement over the performance of the single variant Bad.F6Pkt H548N (Figure S6).

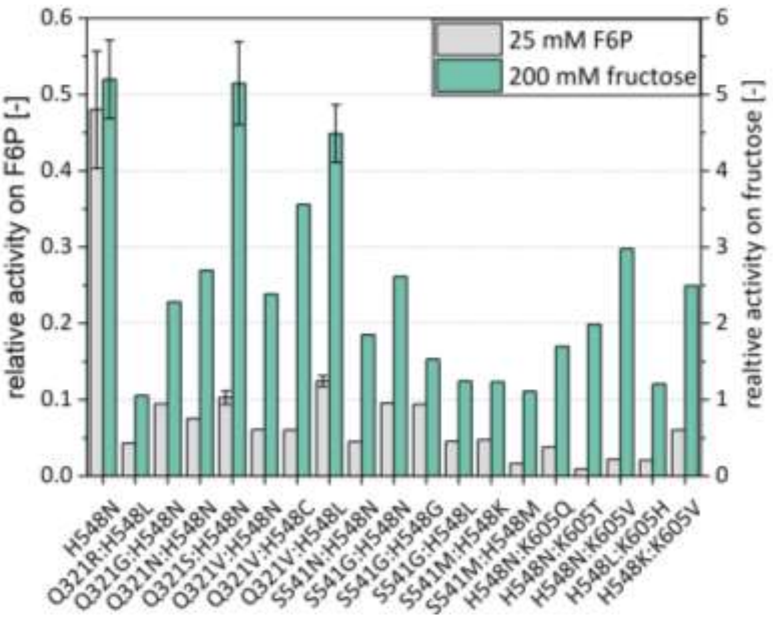

Figure S6 Relative D-fructose and fructose 6-phosphate (F6P) activity of Bad.F6Pkt double mutants selected from focused libraries. After identification by screening of focused double mutation libraries, beneficial variants were produced in shake flasks, purified and their specific activity was determined at pH 6.5, 37 °C in the presence of 50 mM inorganic phosphate. Relative activity was determined as the ratio of the specific activities of the mutant and wild-type enzymes. Data with error bars are presented as mean and standard deviation (n ≥ 3). Data without error bars represent the result of a single measurement.

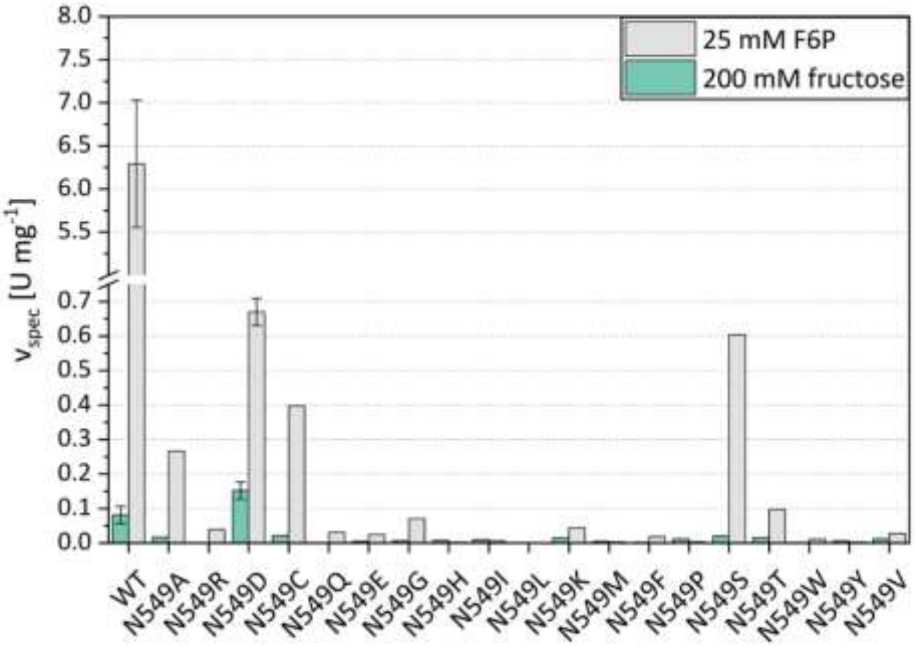

Figure S7 Specific activity of Bad.F6Pkt N549 single variants on D-fructose and the natural substrate fructose 6-phosphate (F6P). WT – wild type. Protein expression was carried out in shake flasks at 25 °C for 24 h using auto-induction medium. Activity of purified enzyme variants was measured at 37 °C and pH 6.5 in the presence of 50 mM inorganic phosphate. Data with error bars are presented as mean and standard deviation of at least two biological replicates. Data without error bars represent the result of a single measurement.

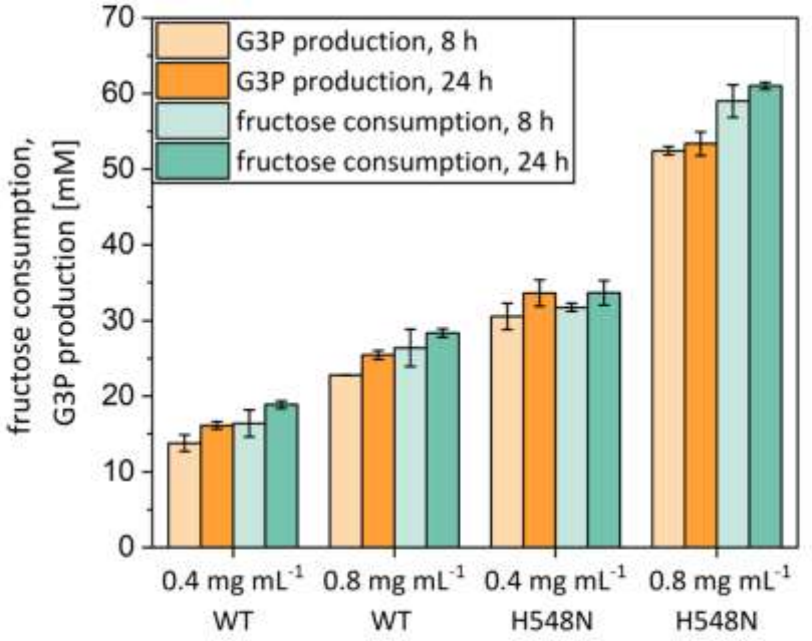

Figure S8 Fructose consumption and G3P production with ATP regenerated from the C_6 ketose using either Bad.F6Pkt wild type (WT) or mutant H548N at varying concentrations. G3P was determined by a photometric assay, whereas fructose was quantified by HPLC analysis. Experiments were carried out at pH 7.0, 37 °C in the presence of 0.8 mM TPP, 4 mM $MgCl_2$ with starting concentrations of 52 mM glycerol as limiting substrate, 100 mM sodium phosphate, 200 mM fructose and 1 mM ADP. Data represent mean and deviation of biological duplicates.

Supporting Information 3: Additional material and methods

Table S5 Plasmids used in this study.

Plasmid	Short description	Source
pET-28a(+)	*ori f1*, Kan^R, T7 promoter	Novagen™
pET28a_Bad.f6pkt	pET-28a(+) carrying *f6pkt* gene from *Bifidobacterium adolescentis* ATCC 15703	This study
pET28a_Bb.pkt	pET-28a(+) carrying *xfp* gene from *Bifidobacterium breve* DSM 20213	This study
pET28a_Bb.xfp	pET-28a(+) carrying *xfp* gene from *Bifidobacterium breve* strain 203	[6]
pET28a_Ca.xfp	pET-28a(+) carrying *xfp* gene from *Clostridium acetobutylicum* ATCC 824	[7]
pET28a_Ps.lrhi	pET-28a(+) carrying *lrhi* gene from *Pseudomonas stutzerii*	This study
pET28a_Gg.der	pET-28a(+) carrying gene from *Gallus gallus*	This study
pET28a_Gs.ackA	pET-28a(+) carrying *ackA* gene from *Geobacillus stearothermophilus* DSM 22	This study
pET28a_Cs.glpK	pET-28a(+) carrying *glpK* gene from *Cellulomonas sp.* NT3060	This study

Table S6 Primers used for plasmid construction. *with pET-28(a)+ homologues regions. Sequence of homologues regions given in lowercase letters.

Primer	Sequence 5' – 3'	Application
157	CATATGGCTGCCGCGCGGCACCAGGCCGCTGCTGTG	amplification of pET-28(a)+ backbone
159	GAATTCGAGCTCCGTCGACAAGCTTGCGGCCGCACTC	
604	ctggtgccgcgcggcagccatatgACG AGT CCT GTT ATT GGC ACC C	amplification of *Bad.f6pkt* *
605	tgtcgacggagctcgaattcTTACTCGTTATCGCCAGCGGTTG	
606	ctggtgccgcgcggcagccatatgACAAATCCTGTTATTGGCACC CCG	amplification of *Bb.pkt* *
607	tgtcgacggagctcgaattcTTACTCGTTGTCGCCTGCG GT	
637	ctggtgccgcgcggcagccatatgGCAAAAGTGTTAGCCATTAATGCG	amplification of *Gs.ackA* *
638	tgtcgacggagctcgaattcCTACAAATTCGCCAGCCGCATG	
901	cctggtgccgcgcggcagccatATGGAGCCGGACCTCAGC	amplification of *Gg.der* *
962	agcttgtcgacggagctcgaattcaTTAGGAGACGAGAAATCCCCCG	
1217	ATGGCCGACTACGTTCTCG	amplification of *Cs.glpK*
1218	TTACTGCTCCACGTCCTCG	
1219	ACGAGGACGTGGAGCAGTAAgaattc gagctccgtcgacaagc	amplification of pET-28(a)+ backbone with Cs.*glpK*-homologues regions
1220	ATGGCGAGAACGTAGTCGGCcatatg gctgccgcgcgg	

Table S7 Expression conditions of his-tagged proteins from pET28a-derived vector. In an initial round of screening, eight different conditions were evaluated to identify the one yielding the highest protein expression level.

Protein	*E. coli* host strain	Expression conditions
Bad.F6Pkt	Rosetta(DE3) plysS	auto-induction medium, at 25 °C for 24 h
Bb.Pkt	Rosetta(DE3) plysS	auto-induction medium, at 25 °C for 24 h
Bb.Xfp	BL21(DE3)	auto-induction medium, at 25 °C for 24 h
Ca.Xfp	Rosetta(DE3) plysS	LB medium, at 37 °C for 3 h
Ps.LhrI	Rosetta(DE3) plysS	LB medium, at 25 °C for 20 h
Gg.DER	Rosetta(DE3) plysS	LB medium, at 25 °C for 20 h
Gs.AckA	BL21(DE3)	LB medium, at 25 °C for 20 h
Cs.GlpK	Rosetta(DE3) plysS	LB medium, at 25 °C for 20 h

Table S8 Design of a focused library to study the interaction between positions H548 and Q321. A residue whose substitution in previous experiments resulted in increased fructose activity is referred to as a 'positive single mutant'.

position	allowed residues	chemical properties of allowed residue	other criteria
H548	K - lysine	positive charge	
	C - cysteine	hydrophilic, reactive with –OH groups	positive single mutant
	E - glutamate	negative charge	positive single mutant
	N - asparagine	hydrophilic	best single mutant
	L - leucine	hydrophobic	positive single mutant
	Y - tyrosine	aromatic, big	positive single mutant
Q321	H - histidine	positive charge	
	R - arginine	positive charge, big	
	Y - tyrosine	aromatic, big	
	W - tryptophan	aromatic, big	
	P - proline	small, immino group, rigid	
	N - asparagine	hydrophilic, small	
	S - serine	hydrophilic, small	
	V - valine	hydrophobic, small	positive single mutant
	G - glycine	small	positive single mutant

Table S9 Design of a focused library to study the interaction between positions H548 and R442. A residue whose substitution in previous experiments resulted in increased fructose activity is referred to as a 'positive single mutant'.

position	allowed residues	chemical properties of allowed residue	other criteria
H548	K - lysine	positive charge	
	G - glycine	small	positive single mutant
	E - glutamate	negative charge	positive single mutant
	N - asparagine	hydrophilic	best single mutant
	L - leucine	hydrophobic	positive single mutant
	Y - tyrosine	aromatic, big	positive single mutant
	M - methionine	hydrophobic, big	positive single mutant
R442	H - histidine	positive charge	
	G - glycine	small	
	D - aspartate	negative charge	
	N - asparagine	hydrophilic	
	L - leucine	hydrophobic	
	Y - tyrosine	aromatic, big	
	M - methionine	hydrophobic, big; structurally close to R but without charge	

Table S10 Design of a focused library to study the interaction between positions H548 and S541. A residue whose substitution in previous experiments resulted in increased fructose activity is referred to as a 'positive single mutant'.

position	allowed residues	chemical properties of allowed residue	other criteria
H548	K - lysine	positive charge	
	G - glycine	small	positive single mutant
	E - glutamate	negative charge	positive single mutant
	N - asparagine	hydrophilic	best single mutant
	L - leucine	hydrophobic	positive single mutant
	Y - tyrosine	aromatic, big	positive single mutant
	M - methionine	hydrophobic, big	positive single mutant
S541	K - lysine	positive charge	
	G - glycine	small	
	E - glutamate	negative charge	
	N - asparagine	hydrophilic	best single mutant in this position
	V - valine	hydrophobic, smaller	
	M - methionine	hydrophobic, big	
	Y - tyrosine	aromatic, big	

Table S 11 Design of a focused library to study the interaction between positions H548 and K605. A residue whose substitution in previous experiments resulted in increased fructose activity is referred to as a 'positive single mutant'.

position	allowed residues	chemical properties of allowed residue	other criteria
H548	K - lysine	positive charge	
	C - cysteine	Hydrophilic, reactive with –OH groups	positive single mutant
	E - glutamate	negative charge	positive single mutant
	N - asparagine	hydrophilic	best single mutant
	L - leucine	hydrophobic	positive single mutant
	Y - tyrosine	aromatic, big	positive single mutant
K605	H - histidine	positive charge	
	E - glutamate	negative charge	
	V - valine	hydrophobic, small	positive single mutant; beneficial in transketolase regarding activity on non-phosphorylated substrate [8]
	M - methionine	hydrophobic, big	
	T - threonine	hydrophilic	best single mutant in this position
	Q - glutamine	hydrophilic	beneficial in transketolase regarding activity on non-phosphorylated substrate [9]

References

(1) Sievers, F.; Wilm, A.; Dineen, D.; Gibson, T. J.; Karplus, K.; Li, W.; Lopez, R.; McWilliam, H.; Remmert, M.; Söding, J.; Thompson, J. D.; Higgins, D. G. Fast, Scalable Generation of High-quality Protein Multiple Sequence Alignments Using Clustal Omega. *Molecular Systems Biology* **2011**, *7* (1), 539. https://doi.org/10.1038/msb.2011.75.

(2) Madeira, F.; Madhusoodanan, N.; Lee, J.; Eusebi, A.; Niewielska, A.; Tivey, A. R. N.; Lopez, R.; Butcher, S. The EMBL-EBI Job Dispatcher Sequence Analysis Tools Framework in 2024. *Nucleic Acids Research* **2024**, *52* (W1), W521–W525. https://doi.org/10.1093/nar/gkae241.

(3) Robert, X.; Gouet, P. Deciphering Key Features in Protein Structures with the New ENDscript Server. *Nucleic Acids Research* **2014**, *42* (W1), W320–W324. https://doi.org/10.1093/nar/gku316.

(4) Westbrook, J. D.; Shao, C.; Feng, Z.; Zhuravleva, M.; Velankar, S.; Young, J. The Chemical Component Dictionary: Complete Descriptions of Constituent Molecules in Experimentally Determined 3D Macromolecules in the Protein Data Bank. *Bioinformatics* **2015**, *31* (8), 1274–1278. https://doi.org/10.1093/bioinformatics/btu789.

(5) Suzuki, R.; Katayama, T.; Kim, B.-J.; Wakagi, T.; Shoun, H.; Ashida, H.; Yamamoto, K.; Fushinobu, S. Crystal Structures of Phosphoketolase. *Journal of Biological Chemistry* **2010**, *285* (44), 34279–34287. https://doi.org/10.1074/jbc.M110.156281.

(6) Suzuki, R.; Kim, B.-J.; Shibata, T.; Iwamoto, Y.; Katayama, T.; Ashida, H.; Wakagi, T.; Shoun, H.; Fushinobu, S.; Yamamoto, K. Overexpression, Crystallization and Preliminary X-Ray Analysis of Xylulose-5-Phosphate/Fructose-6-Phosphate Phosphoketolase from *Bifidobacterium Breve*. *Acta Crystallogr F Struct Biol Cryst Commun* **2010**, *66* (8), 941–943. https://doi.org/10.1107/S1744309110023845.

(7) Wagner, N.; Bade, F.; Straube, E.; Rabe, K.; Frazão, C. J. R.; Walther, T. In Vivo Implementation of a Synthetic Metabolic Pathway for the Carbon-Conserving Conversion of Glycolaldehyde to Acetyl-CoA. *Front. Bioeng. Biotechnol.* **2023**, *11*. https://doi.org/10.3389/fbioe.2023.1125544.

(8) Hibbert, E. G.; Senussi, T.; Costelloe, S. J.; Lei, W.; Smith, M. E. B.; Ward, J. M.; Hailes, H. C.; Dalby, P. A. Directed Evolution of Transketolase Activity on Non-Phosphorylated Substrates. *Journal of Biotechnology* **2007**, *131* (4), 425–432. https://doi.org/10.1016/j.jbiotec.2007.07.949.

(9) Strafford, J.; Payongsri, P.; Hibbert, E. G.; Morris, P.; Batth, S. S.; Steadman, D.; Smith, M. E. B.; Ward, J. M.; Hailes, H. C.; Dalby, P. A. Directed Evolution to Re-Adapt a Co-Evolved Network within an Enzyme. *Journal of Biotechnology* **2012**, *157* (1), 237–245. https://doi.org/10.1016/j.jbiotec.2011.11.017.
